# Non invasive subsurface imaging to investigate the site evolution of Machu Picchu

**DOI:** 10.1038/s41598-023-43361-x

**Published:** 2023-09-25

**Authors:** Nicola Masini, Gerardo Romano, Dominika Sieczkowska, Luigi Capozzoli, Daniele Spizzichino, Francesco Gabellone, Jose Bastante, Manuela Scavone, Maria Sileo, Nicodemo Abate, Claudio Margottini, Rosa Lasaponara

**Affiliations:** 1 CNR-Institute of Heritage Science, C.da S. Loya, 85050 Tito Scalo, Italy; 2 University of Bari, Bari, Italy; 3 Silesian University of Technology, Gliwice, Poland; 4 CNR-Institute of Methodologies for Environmental Analysis, C.da S. Loya, 85050 Tito Scalo, Italy; 5 ISPRA, Geological Survey of Italy, Rome, Italy; 6 CNR, Nanotech, Lecce, Italy; 7 Peruvian Ministry of Culture-Directorate of Culture of Cusco, Programa de Investigaciones Arqueologicas e Interdisciplinarias en el Santuario Historico de Machupicchu (PIAISHM), Cusco, Peru; 8 UNESCO Chair at Florence University, Firenze, Italy

**Keywords:** Environmental sciences, Engineering

## Abstract

The construction history of a site is partially preserved underground and can be revealed through archaeological investigations, including excavations, integrated with earth observation (EO) methods and technologies that make it possible to overcome some operational limits regarding the areal dimensions and the investigation depths along with the invasiveness of the excavations themselves. An integrated approach based on EO and archaeological records has been applied to improve the knowledge of Machu Picchu. The attention has been focused on the first construction phase of Machu Picchu, and for this reason the investigations were directed to the imaging and characterization of the subsoil of the Plaza principal, considered the core of the whole archaeological area. Archaeological records and multiscale remote sensing (including satellite, UAS, and geophysical surveys) enabled the identification and characterization of the first construction phase of the site, including the preparation phases before building Machu Picchu. The interpretative hypothesis on the constructive history of Machu Picchu started from the identification and use of the quarry, followed by the planification and set of the drainage systems and by the next steps based on diverse reshaping phases of what would be the central plaza.

## Introduction

### Motivation

Climate and environment, cultural and religious motivations along with the availability of resources in terms of food, water, and construction material, have been the main factors that most influenced the development of civilizations and settlements. On the other hand, over time, the shortage of resources and the need to face natural risks fostered efficient strategies of adaptation thus transforming problems in opportunities and driving the development of technologies and techniques.

This is the case of Machu Picchu (known as *llaqta* in Quechua) an Inca site (in Peru) sit up on an earthquake fault over an inaccessible mountain, with steep slopes, landslides, and abundant rainfall (nearly 2000 mm per year) which posed critical drainage challenges, successfully faced by the Inca, developing advanced engineering techniques still today not fully understood^1^.

Findings from two stratigraphic trial trenches, conducted in the Plaza Principal (see Figs. 2a–c, 6, along with section D *Archaeological record* in Supplementary Information (SI)), opened new research questions about the construction phases of Machu Picchu. To contribute to answer these questions and to better understand how the Machu Picchu site was before becoming the Inca sanctuary and citadel, multiscale and multisource Earth Observation (EO) methods were adopted for the imaging and characterization of the subsoil of the Plaza principal, considered the core of the whole archaeological area.

The interpretative hypothesis of the constructive history of Machu Picchu was formulated on the basis of the EO broad subsurface imaging integrated with architectural evidence and findings from the archaeological excavations (made from its scientific discovery up today) which enabled this understanding.

The methodological approach herein proposed enabled us to investigate the whole area of the Plaza Principal (which cannot be excavated), integrating and enhancing: (i) local and direct information derived from archaeological excavations, (ii) with wider but indirect information from Earth Observation.

### Status of research

**Figure 1:**
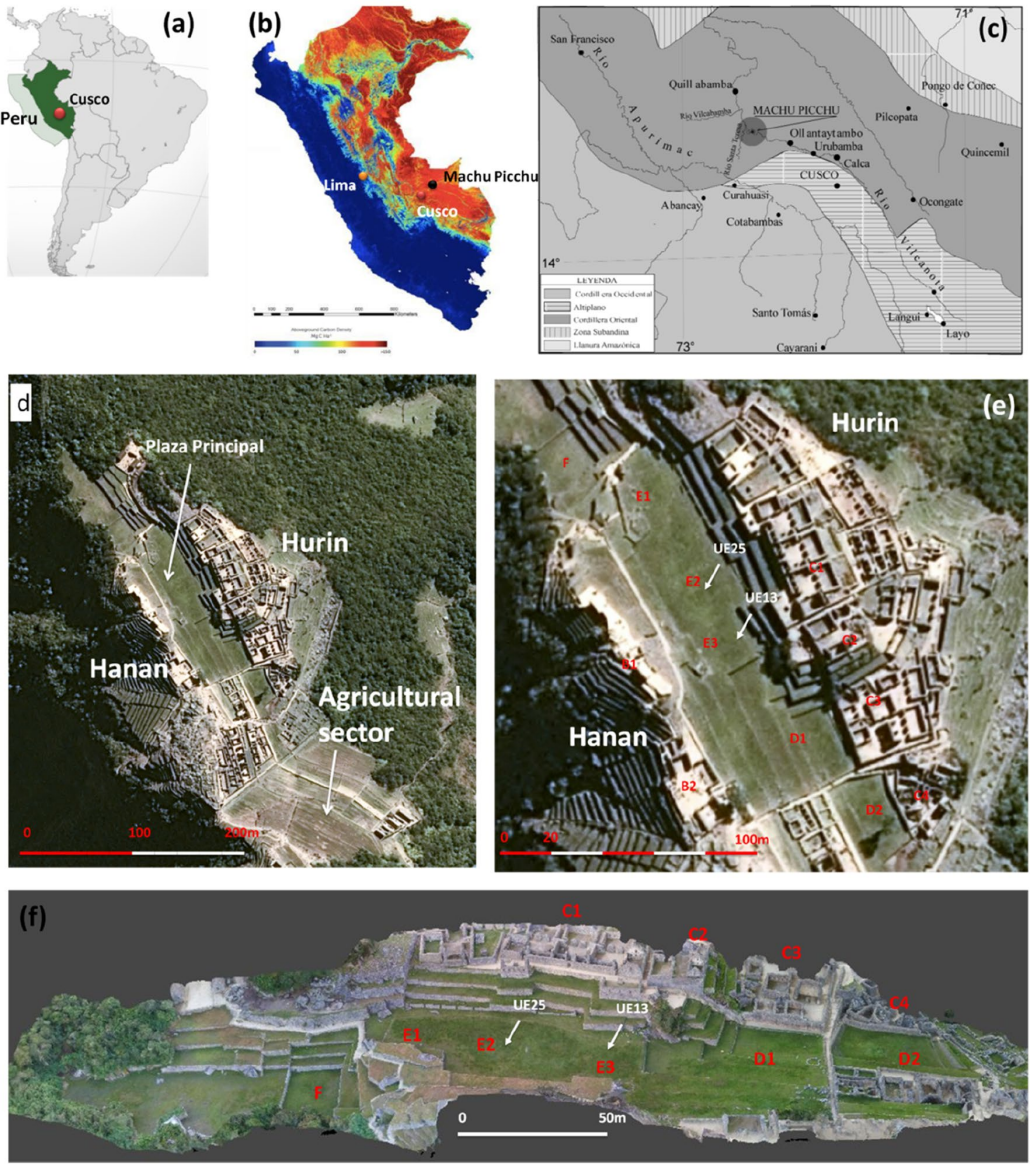
(**a**–**c**) Southern America and Peru: geographic and geological location of Cusco and the *llaqta* of Machu Picchu settled on the Eastern Cordillera of the Andes and surrounded by the Western Cordillera, the Plateau (where Cusco is located), Amazon plain, and Sub-Andean area. (**d**) GeoEye satellite-based map of the *llaqta* of Machu Picchu which shows the main sectors of the site: the agricultural sector at the South and the urban sector in North, divided into two subsectors, the Hanan (to the west) and Hurin (to the east) separated by the 'Plaza Principal'. (**e**) Zoom of image 1d focused on the Plaza Principal (1e); (**f**) 3D model obtained from the UAS-based aerial photogrammetry. In (**e**,**f**), the letters indicate Intihuatana (B1), Sacred square, including the Main Temple and the Three Window Temple (B2), the building complex known as the ‘Three Portals’ (C1), residential area (C2), the Condor Temple (C3), *Plaza Principal* (E1, E2, E3), some terraces known as *andenes* (D1) and second Plaza (D2): Northwest *andenes* (F). UE25 refers to the two excavation units in *Plaza Principal*.

Machu Picchu (Lat. 13° 09′ S, Long. 72° 31′ W), designated by Unesco as World Heritage Site in 1983, is located on the top of a graben-like structure at 2.430 m.a.s.l., in the high Eastern Cordillera called Vilcabamba of the Peruvian Andean chain, at 500 m above the Vilcanota river, also known as Urubamba (Fig. 1a,b).

The Machu Picchu granitoid pluton, forming part of the larger "Quillabamba granite", is one of a series of plutons intruded along the axial zone of the high Eastern Cordillera Permo-Liassic rift system (Fig. 1c), including a variety of rock types, dominantly granites and granodiorites^2,3^.

The site sits up on an inaccessible mountain, between the top of a rocky outcrop named Machu Picchu (which means "Old Mountain" in Quechua) and a natural backdrop known as Huayna Picchu ("Young Mountain"). The area is characterized by essential faults and steep slopes, strongly threatened by landslides and erosion due to frequent and abundant (yearly around 2000 mm) tropical rainfalls^4,5^, that drove the development of advanced engineering techniques^6^. Since its scientific discovery in 1911 by Hiram Bingham^7,8^ questions remain as to the choice of this challenging setting and the particulars of its engineering; although, some important advances in the lasts three decades in the knowledge of Inca masonry building techniques^9,10^, drainage infrastructures, and terraced agricultural systems^6,11^. In particular, puzzling research questions regards the construction phases of the monument, including the quarries and all the preparatory phases, before bulding walls, terraces, temples and houses^6,9, 10^.

**Figure 2:**
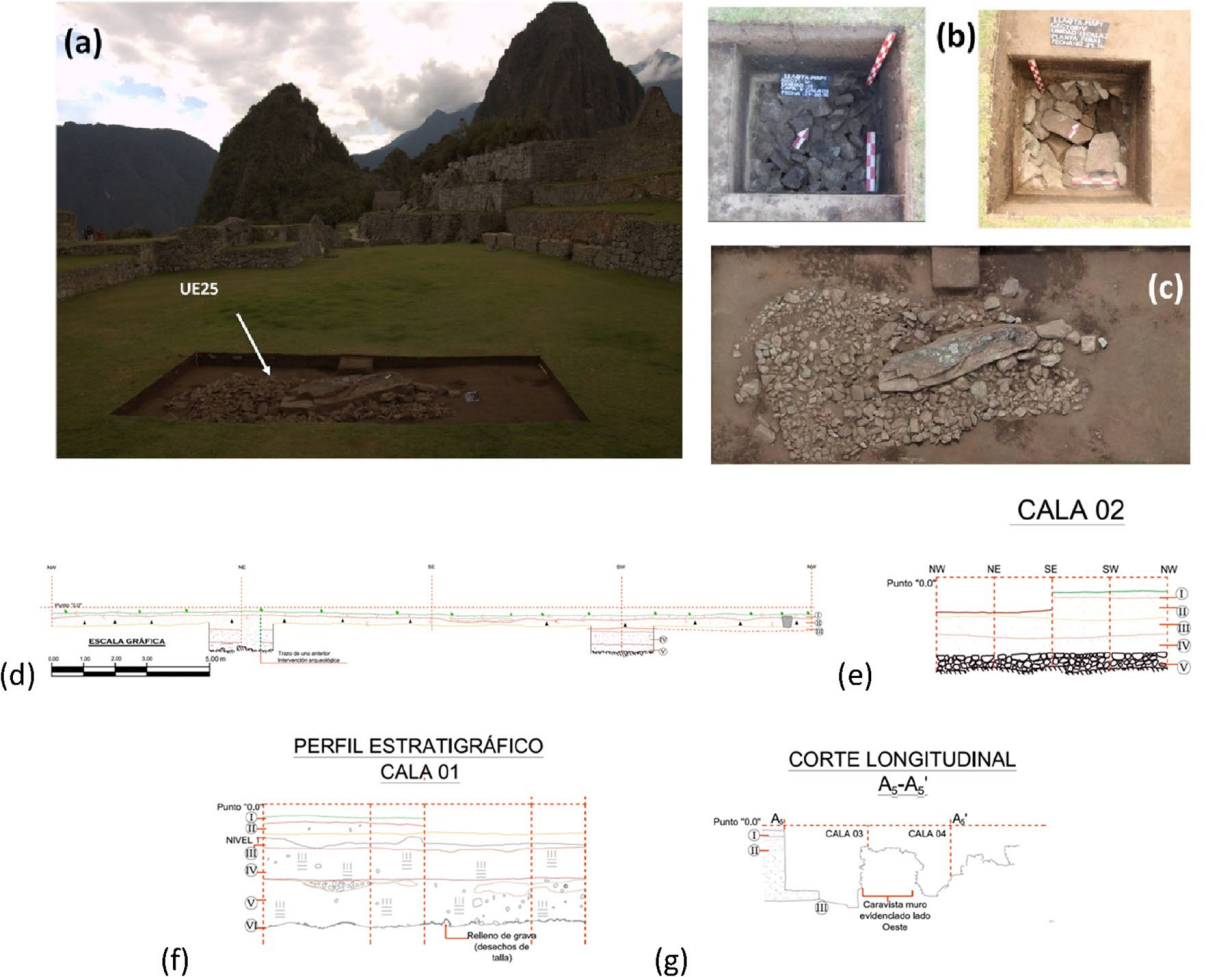
(**a**) Detail of the *Plaza Principal* with the location of UE25; (**b**) Detail of UE13; (**c**) Detail of UE25; (**d**) stratigraphic profile of UE13 with all trenches marked and (**e**) details for the probe 02 (cala in spanish) of UE13; (**f**) stratigraphic profile of trench 01 of UE25; (**g**) longitudinal cut of trenches 03 an 04 of UE25. (**d**–**f**) (credits for PIAISHM archives) The name UE comes from the Spanish name Unidad de Excavación (Archaeological Unit) for which the abbreviation is UE. It means a single excavation unit, or a single area subjected to excavation. As we did not want to interfere with internal terminology at any stage, we decided to use the same nomenclature to avoid problems around naming the same phenomena.

Radiocarbon dating^12,13^ places the initial settlement at the beginning of the fifteenth century and its abandonment most probably after the Spanish invasion and conquest in the mid-sixteenth century. Bingham returned in 1912 to direct the first archaeological excavations that, along with following investigations, highlighted that Machu Picchu was an administrative, political, and religious center, core of a network of satellite sites, extremely important in the cultural interaction between the Amazonian selva, Andean highlands, and coastal shoreline (known as “vertical archipelago”^14,15^). The Incas were able to access labor from the conquered populations (generally resettled in new areas) who paid a tribute in the form of labour, known as mit’a^16^ (Fig. 2).

## Results

This chapter shows the different time phases identified under the Plaza Principal, ranging from phase 0 ("Phase 0: the drainage basin") before the anthropogenic modifications, to the last phase of the square ("The Plaza in the light of archaeological data"), including intermediate phases relating to the use of the catchment area as a quarry ("The Plaza in the light of archaeological data") and the construction of drainage works ("Preparatory phases: the drainages systems").

### Phase 0: the drainage basin.

**Figure 3:**
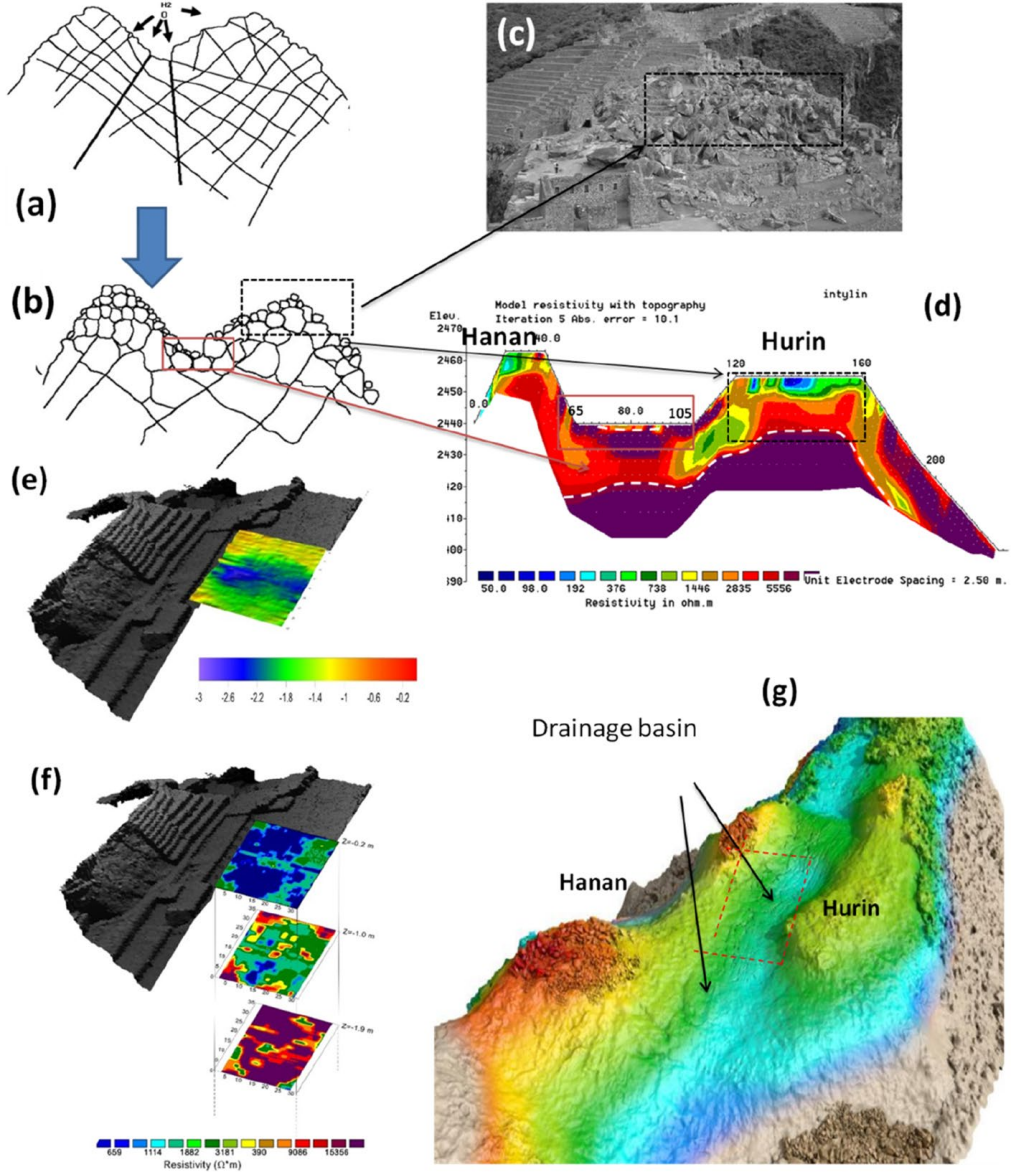
(**a**,**b**) Evolution of granite chaos in two phases: in the first (**a**) rainwater penetrated through fractures and faults, and in the second (**b**) rainwater and gravity separated the granite blocks, thus forming the granite chaos (5); (**c**) Outcrops of granite chaos, at the southwest side close to the Hanan sector; (**d**) ERT profile by Beck et al.^17^ crossing the hilly reliefs of *Hanan*, *Hurin* and the Plaza principal. (**e**) Bedrock surface reconstruction as derived from the GPR survey; (**f**) Geoelectrical depth slices at z = − 0.2 m, Z = − 1.0 m and z = − 1.9 m; (**g**) virtual reconstruction of the drainage basin.

The geomorphological analysis (for additional details see Sect. C and Fig. S12 in SI) suggested that the Plaza Principal is located above a small catchment with its drainage basin placed between the two reliefs (Figs. 1d,e, and 3). This catchment forms part of an impluvium furrow oriented as northwest-southeast, composed of granite and subordinately granodiorite blocks. Prior to any modification, this area comprised small basins with a relatively new drainage network^5^. The site was characterized by fractured granite bedrock as evolution in granite chaos (Fig. 3a–c) resulting from succession of intense precipitations. The considerable abundance of rock, also evident from the Electrical resistivity tomography (ERT) by Best et al. (38), showed in Fig. 3d, provided easily available building material.

The geophysical investigations (see also Sects. B and C in SI) confirmed the geological and geomorphological assumptions on the original shape of the area (Fig. 3). Results from ERT and Ground Penetrating Radar (GPR) located the bedrock at a depth from 2.0 to 3.5 m below the current ground level (Fig. 3e,f). A rounded shaped basin (Fig. 3e,f) was identified from the ERT and this well fitted with the less resistive layers (attributed to the granitic chaos resulting from weathering processes) located between ~ 65 m and ~ 105 m (Fig. 3d) in the more extensive but less detailed (Fig. 3d) survey by Best et al.^17^.

The constraints related to “Phase 0” are the:

- presence of coarse material, including granitic chaos^2^, suitable for both filtering and stabilizing foundation;
- gathering and disposal of rainwater, regularization of the bottom level, and the terrain alignment for future construction.

The advanced Inca hydraulic and geotechnical engineering is clearly evident in the transition from Phase 0 to Phases I and II.

### Phase I: the quarry

**Figure 4:**
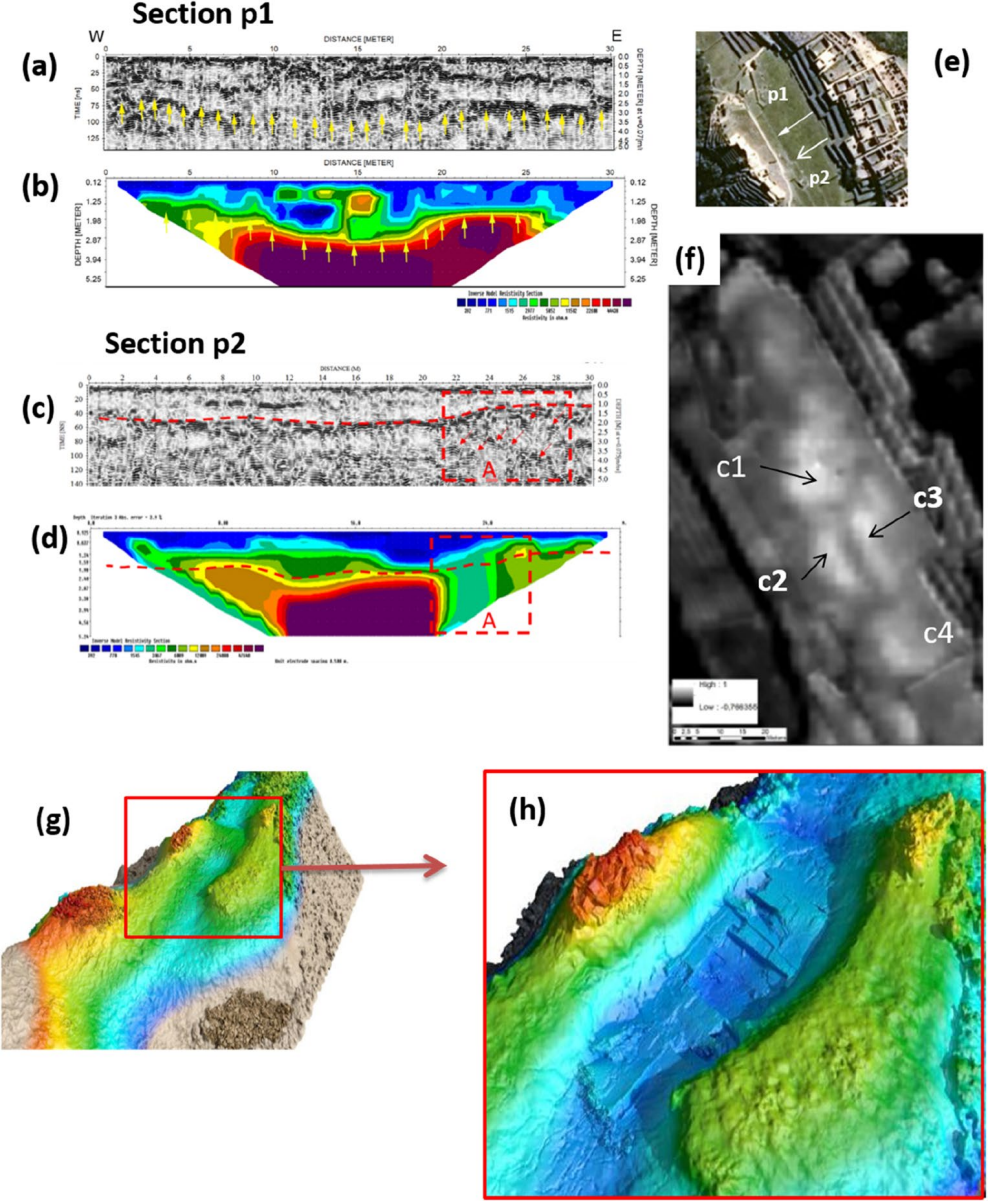
(**a**)-GPR section p1; (**b**) ERT section p1; (**c**) GPR section p2; (**d**) ERT section p2; (**e**) location of sections p1 and p2; (**f**) satellite NDVI map with crop-marks; (**g**,**h**) virtual reconstruction from the phase 0 (the drainage basin) to the phase I (the quarry). The yellow arrows in (3a) indicate the radar reflective surface. The same arrows have been superimposed on the ERT profile (3b), highlighting that the reflective surface roughly matches with the top edge of a resistive body related to granite bedrock.

The first transformation of Machu Picchu is characterized by the quarry activity that reshaped the drainage basin. The consensus is that the quarry was dispersed rock material resulting from erosion between the peaks that survive to the northeast of Plaza Principal and east of Sacred Plaza. Geophysical imaging highlighted that, below the Plaza Principal, the bedrock is characterized by irregular jagged and indented shapes (Fig. 4a,b) thus suggesting the presence of loosely attached large blocks (see also Fig. 8d,e) typical of natural or manufactured fractured rock complex (an ancient rip rap type processing). In the ERT and GPR maps, these areas are identified by interruptions of resistive deep surfaces and reflectors (denoted with dashed red box in Fig. 4c,d), respectively. The granite rocks' jagged and fractured morphology is also visible from the georadar profiles (see Fig. 4e) which evidence the presence of sub-vertical surfaces of the granite rocks, related to quarry extraction.

The geoelectrical depth slices (see Fig. 3f along with Sect. B.1 in SI) provide additional morphological and dimensional details on resistive elements related to the granite blocks. At 2m, less resistive areas with irregular presence were found. These spaces could be associated with the extraction of granite blocks (probably already fractured) and incoherent rocky material. Accordingly, the GPR radargrams (see Fig. 4c) exhibit local reflectors (red arrows) at depths greater than 3 m, referring to natural rock discontinuities linked to fractures or cuts (see also Fig. S10 in SI).

The geophysical surveys found an irregular and complex topography of the bedrock, which corresponds to a heterogeneous soil filling up to the current level of the square. This spatial variation of fill produces changes in moisture content and vegetation growth (the typical crop-marks^18,19^; see also Sect. A in SI), clearly visible from the multispectral remote sensing data (Fig. 4f; and Figs. S4–S6 and Sect. A.3 in SI).

**Figure 5:**
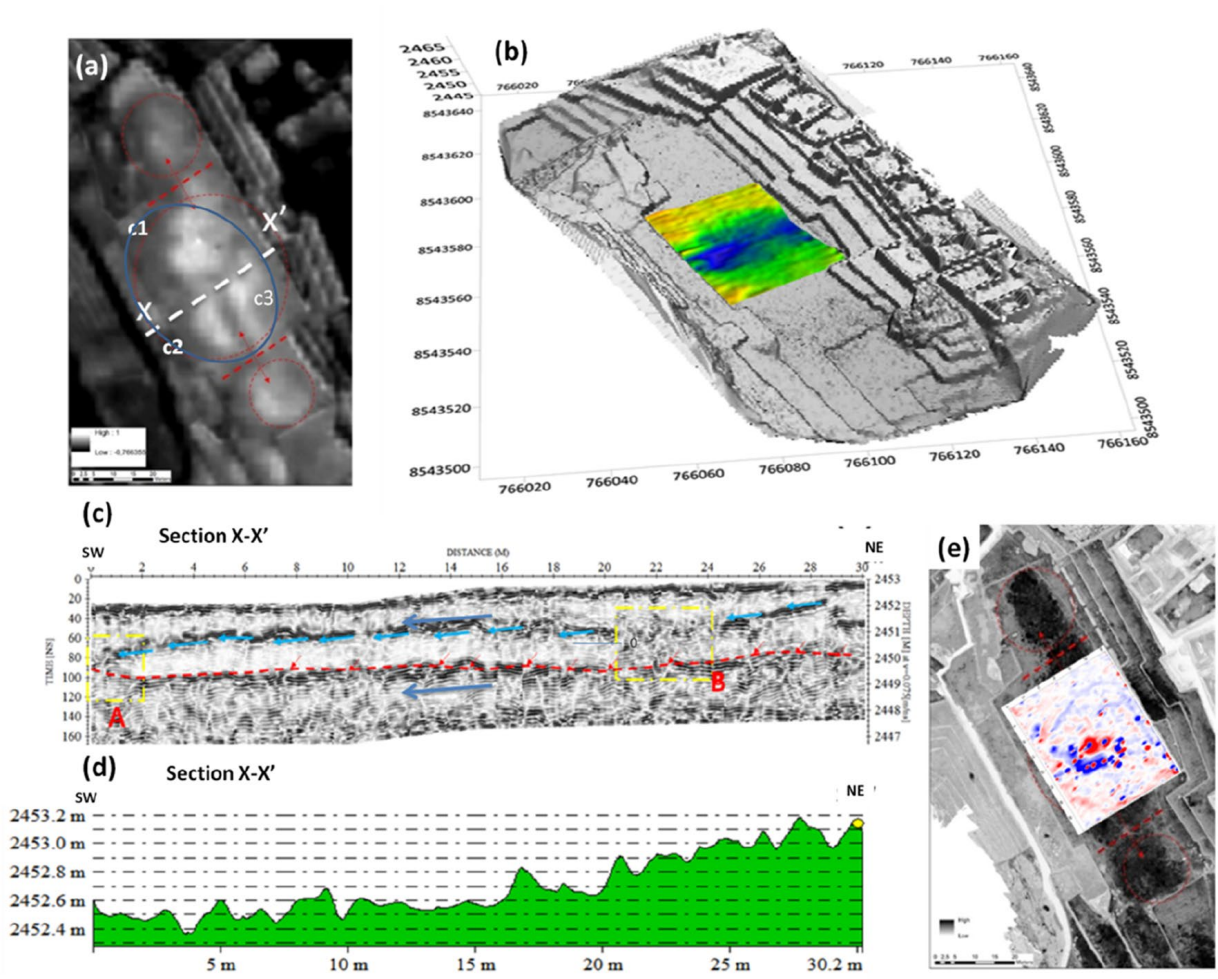
(**a**) Satellite based NDVI map which puts in evidence three crop-marks c1, c2, and c3 related to spatial variations of soil filling; (**b**) GPR imaging overlaid on the 3d model obtained by UAS based photogrammetry; (**c**) GPR section X–X′ crossing the Plaza Principal along the NE–SW; (**d**) Topographic section X–X′; (**e**) Magnetic map.

Results from satellite-based analyses reveal a large crop mark (40 × 32 m, approximately), caused by the differences in fill depths, particularly evident in the NDVI (Normalized Difference Vegetation Index) map (see Fig. 4f; Table S1 in SI). Higher NDVI values (compared to the neighboring areas) are related to greener or healthier vegetation due to higher moisture and terrain depth. These variations in vegetation growth and soil moisture are also visible from the magnetic susceptibility survey in Fig. 5e^20^.

The UAS-based maps show additional smaller crop marks probably related to the reorganization of the quarry over time, later transformed into the Plaza Principal (Sect. A.3, Figs S4–S5b in SI).

The reconstruction in Fig. 3g,h recreates the manner that quarrying modified the catchment basin.

### Phase II: The Plaza

#### Preparatory phases: the drainages systems

The shape of the catchment basin was modified and filled to create the stable foundations for the Plaza Principal. For the Incas, the priority was to design a water drainage system to avoid water infiltration. For example, the Temple of the Sun was affected by deformation and collapses. The GPR 3D image (see Fig. 5b) shows the concavity shape of the bedrock (see also Fig. 8a) which highlights the excellent Inca engineering techniques for the water flow management. The Incas were fully aware of the destructive power of uncontrolled water, so its proper management was always one of the first characteristic elements of the Inca construction. The control of water resource was not only essential on a practical level, but symbolically represented a manifestation of political power^21–23^.

The satellite NDVI map highlights some crop marks (c1, c2, and c3, in Fig. 5a) which help to identify potential drainage collectors (characterized by darker tones, corresponding to lower values of NDVI). The GPR sections topographically corrected using the DTM from UAS photogrammetry show a gentle slope from NE to SW (as shown in the radar section X–X′ in Fig. 5c). In particular, the slope in X–X′ section is around 2.6% at the square level (Fig. 5d) and 6% along the radar reflective layer indicated with light blue and red color in Fig. 4c. This morphological condition represents a good solution for the drainage of surface water. Moreover, the radar section X–X′ also puts in evidence two strong local reflectors (A and B, highlighted by dashed yellow rectangles in Fig. 5c), that interrupt the shallow reflecting layer (indicated with light blue arrows), referable to the presence of drainage structures common in Machu Picchu.

Below this level, there is another reflective layer at a depth greater than 2 m (see red dashed lines in Fig. 5c), interrupted by local reflections reasonably due to natural fractures and/or quarry cuts, defining a ‘two-level’ (anthropogenic and natural) drainage system. The latter guided the water into the large central drainage basin in the NE/SW direction to avoid an excessive and dangerous water load near the north and south walls. To facilitate the evacuation of water, the area was re-shaped (regularizing the bed of the quarry) and filled using stone/waste material, silty sands with gravel, and sandy silt, as confirmed by two archaeological trenches (see Fig. 2a–c; "Introduction").

#### The Plaza in the light of archaeological data

After the reshaping of the hydrographic basin (by quarrying) and the construction of the drainage systems, the Incas’ efforts were addressed to build a space for ceremonial activities. Different phases of soil re-filling and compaction were identified combining geophysical results with the archaeological data^19^, so that the main question to answer is: does each filling layer only a construction phase or correspond to a phase of attendance of the Plaza?

To answer this question, we combined the archaeological records from units UE13 and UE25^19^ (Figs. 1f, 2a–c) with the results from GPR (Fig. 7).

UE25 was excavated to define the original position and dimensions of a sacred monolith, in Quechua known as *wanka* (previously excavated for restoration and reburied^24^ and extremely important because in the Inca worldview, *it* provided for with ceremonies and offerings. This *wanka* is in the central part of the Plaza Principal and stands as a testimony to the ceremonial nature of this space.

**Figure 6:**
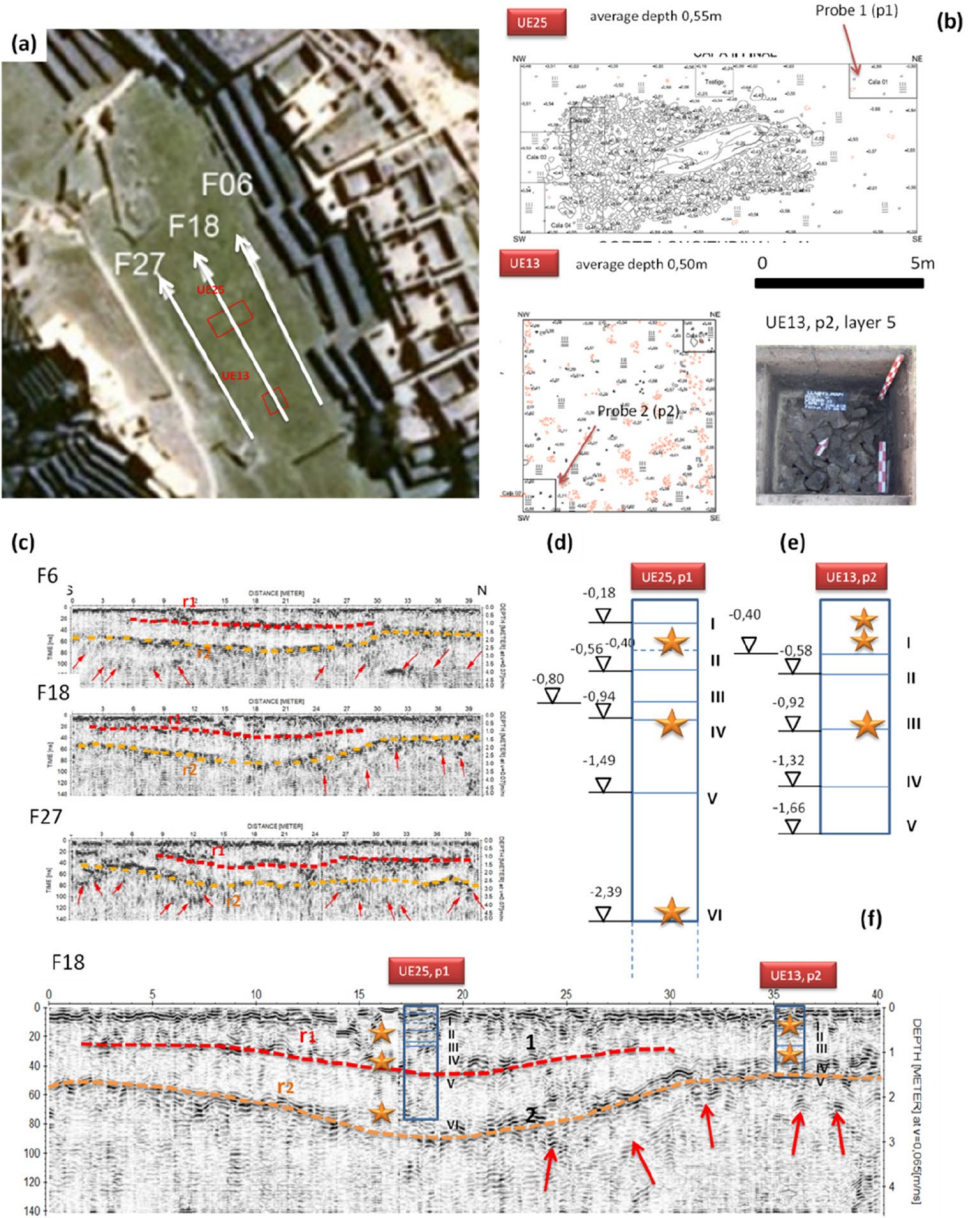
(**a**) Plaza Principal with the location of three GPR sections (F6, F18, F27) and the excavation units UE25 and UE13. (**b**) Excavation units UE25 and UE13: maps with the location of the probes, and a detail of layer 5 of probe 2 of UE13. (**c**) Radargrams F8, F13, and F27. Red and orange dashed lines denote two reflective surface r1 and r2, respectively. Red arrows indicate some local reflectors below r2, referable to granite rock bodies. (**d**,**e**) Stratigraphy of probe 1 of UE25 and probe 2 of UE13, respectively. Orange arrows indicate the presence of cultural material found by the archaeologists. (**f**) Zoom of radargram F18 aimed at compare the archaeological layers with GPR reflective layers.

The excavations revealed two layers at progressive depths of 18 cm and 55 cm (I and II in Figs. 5c, 6b), the expected monolith (horizontally lying), charcoal, and several ceramic sherds from vessels associated with ceremonies, thus confirming that the Plaza was mainly used for ritual activities. From the last excavation level four small probes of size 1–2 m (named 1, 2, 3, 4; see Figs. 2d–f, 6b) were placed to understand the stratigraphy of the Plaza and identify other cultural phases (for additional details see SI, Sect. D, Figs. S14–S15). Probe 1 revealed four layers (III to VI, in Fig. 6d) characterized by silty-sandy soil with different colors and types, whose top surfaces are located at progressive depths of 80 cm, 94 cm, 1.49 m and to 2.40 m. The last layer was composed of lithic fragments, residues of quarrying and stone cutting activities, placed to fill the fractures and interstices between granite blocks^19^. The fragments packed around the foundation stabilized the monolith in an upright position. Three GPR sections conducted on the center of the Plaza Principal (F6, F18, F27, see Fig. 6) exhibit two reflective surfaces, named r1 and r2. The first one, almost horizontal (highlighted with red dashed lines) is 0.80–1.20 m in depth. The second deeper layer (highlighted with orange dashed line) is characterized by a curved shape in the middle and two horizontal sections at both ends, following the form of the underlying catchment area. Below it, several local reflectors (marked with red arrows in Fig. 6) are visible. The comparison between F18 radargram (crossing unit UE25) and the archaeological layers highlights a correspondence between the georadar reflective surface r1 and the interface between the archaeological layers III and IV. The top of the archaeological layer VI (at a depth of 2.40) is close to the georadar reflective surface r2, at a progressive depth of 2.90 m, reasonably caused by the granite bedrock. The difference of half a meter between the top of layer VI and r2 is probably due to a layer of pebbles and lithic fragments. Both the georadar and archaeological layering suggest that at least two human occupation phases characterized the central part of the Plaza. In summary, the only significant cultural layer closely related to the GPR data were lithic elements from monolithic processing, but they were probably not closely related to terrace construction. Only the sand fill may have played a stabilizing role, which is clearly illustrated in probes in form of trenches (calas) 1, 3 and 4 in Fig. 2d–f.

Unit U13, located on the SE edge of the Plaza, revealed two layers (I e II, at progressive depths of 25 cm and 50 m, respectively) composed of silt mixed with gravel and cultural material including decorated ceramic fragments, circular pendants, and stone hammers. To establish the stratigraphy, two probes p1 and p2 were done. The latter, 1.65 m deep, revealed three layers (III to V, in Fig. 6) located at progressive depths of 90 cm, 1.32 m and to 1.65 m, made up by gravels and pebbles for the drainage of rainwater runoff towards the surface of the deeper rocks. This suggested that before building the Plaza, the Incas stabilized the lower platforms with particular attention to water drainage. Such use is clearly evident in the stratigraphic profile of Fig. 2d–f where the NW probe in layer V is characterized by a fill of fine lithic material. This is a structural element of the terraces and evidently the last layer (after the sand layer) which helped control any hydrological movements.

Comparing radargram F18 with the archaeological layers, it is possible to observe a correspondence between the georadar reflective surface r1 with the interface between the archaeological layers III and IV, and between the georadar reflective surface r2 with the top of the archaeological layer V.

Therefore, comparing the archaeological stratigraphy with georadar reflective layering, it is possible to argue at least three soil filling phases.

The question is, are these filling phases only designed to set soil platforms to ensure adequate geotechnical and drainage characteristics of the Plaza, or do correspond (at least 2 out of 3) to diverse phases of attendance of the Plaza. In other words, is there a more ancient plaza under the current one?

From the georadar sections F6 and F18 it is possible to observe that the deeper reflective layer r2 has a mixtilinear shape with a concavity in the center and two horizontal planes. This shape may be a result of the reshaping of the granite bedrock of the water catchment during the quarrying phase. The regular shape of the reflective layer is also due to the presence of crushed stone, lithic fragments, and pebbles placed to fill the fractures and spaces between granite blocks.

The presence of cultural material at various depths, among which are very deep and very close to r2, along with the integration of archaeological and geophysical data, suggest at least two phases of construction of the Plaza Principal.

The first phase could be related to a so-called sunken plaza (*plaza hundida* in Spanish) below 2.5 m from the current surface and smaller than the Plaza Principal. This type of Plaza is usually bounded on all four sides and set below the level of the surrounding *andenes*. Similar spaces are found within the park at Chachabamba, Phuyupatamarca, or Qantupata where they are interpreted as having a ceremonial function.

**Figure 7:**
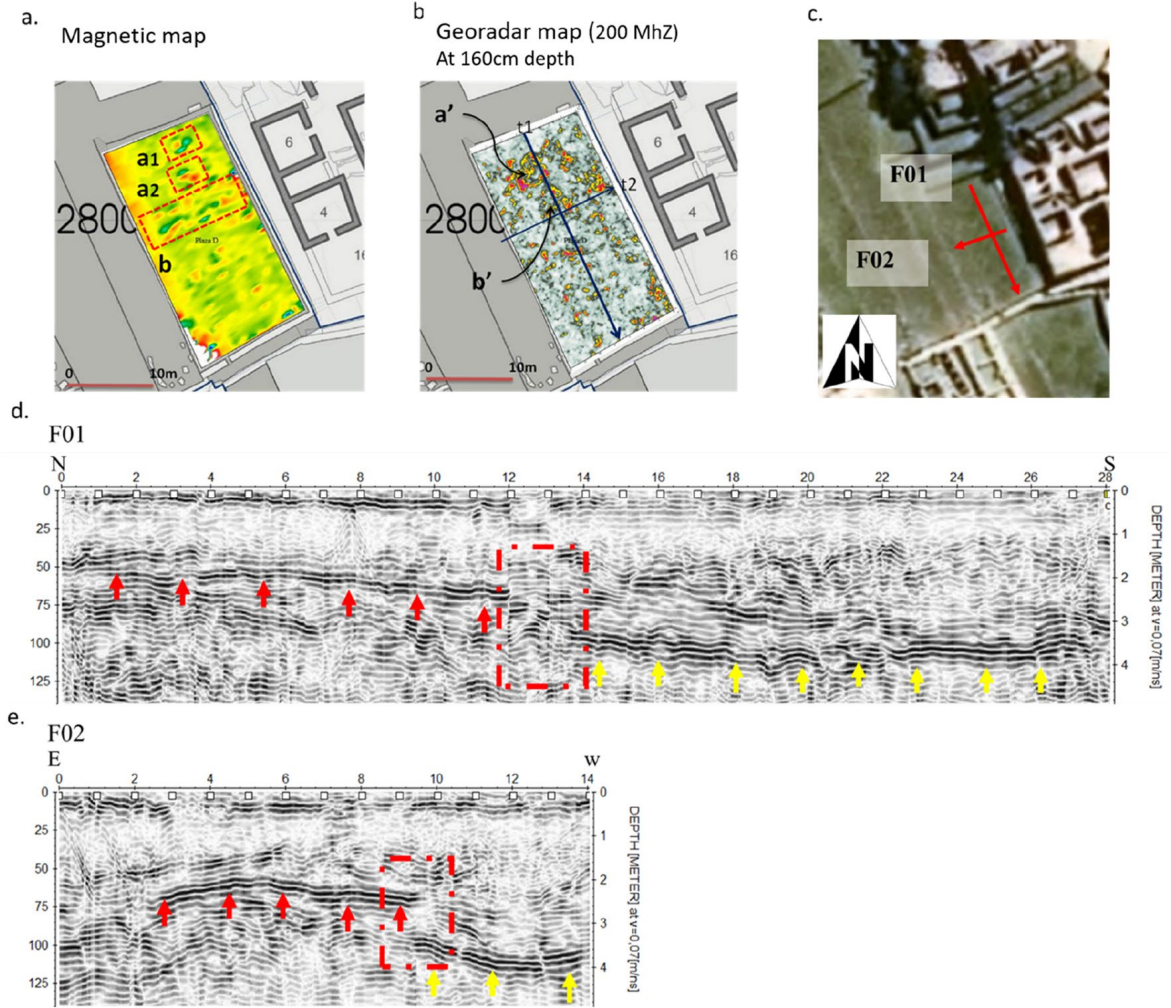
Plaza D1. Geophysical results revealing a two-step construction phase. (**a**) Magnetic map; (**b**) GPR depth-slice map at 1.60 m depth; (**c**) location of the two radargrams F01 (**d**) and F02 (**e**) that reveal the two distinct and overlapped construction phases.

Subsequently, the filling process of the Plaza Principal continued, reaching its current size and shape. In this respect, another question arises: did this filling process occur in a single phase or in more than one? The presence of a strongly reflective surface (at a depth of about 1 m) and the presence of cultural material indicates that between the plaza *hundida* phase and the current Plaza Principal, there was an intermediate construction phase. The georadar profiles and the geomagnetic maps (Fig. 7) identified various fill phases in the southeast of the Plaza Principal, in sector D1 (see also Fig. 1e,f). GPR evidences the presence of a step of the *andenes* covered with earth to create a small plaza (Fig. 7c,d). This multi-stage construction process reveals an approach to the creation of the ceremonial space, as will be explained in more detail in the discussion ("Discussion").

## Discussion

Two stratigraphic trial trenches, conducted in the Plaza Principal (see "Introduction", "The Plaza in the light of archaeological data", Figs. 1f, 2a,b, Fig. 6; along with Figs. S13–S15 in SI), opened new research questions about the construction phases of Machu Picchu. To contribute to answer these questions, non-invasive EO surveys were conducted in the entire Plaza Principal and its adjacent *andenes* thus revealing various construction preparatory phases.

### From the drainage basin to quarry

**Figure 8:**
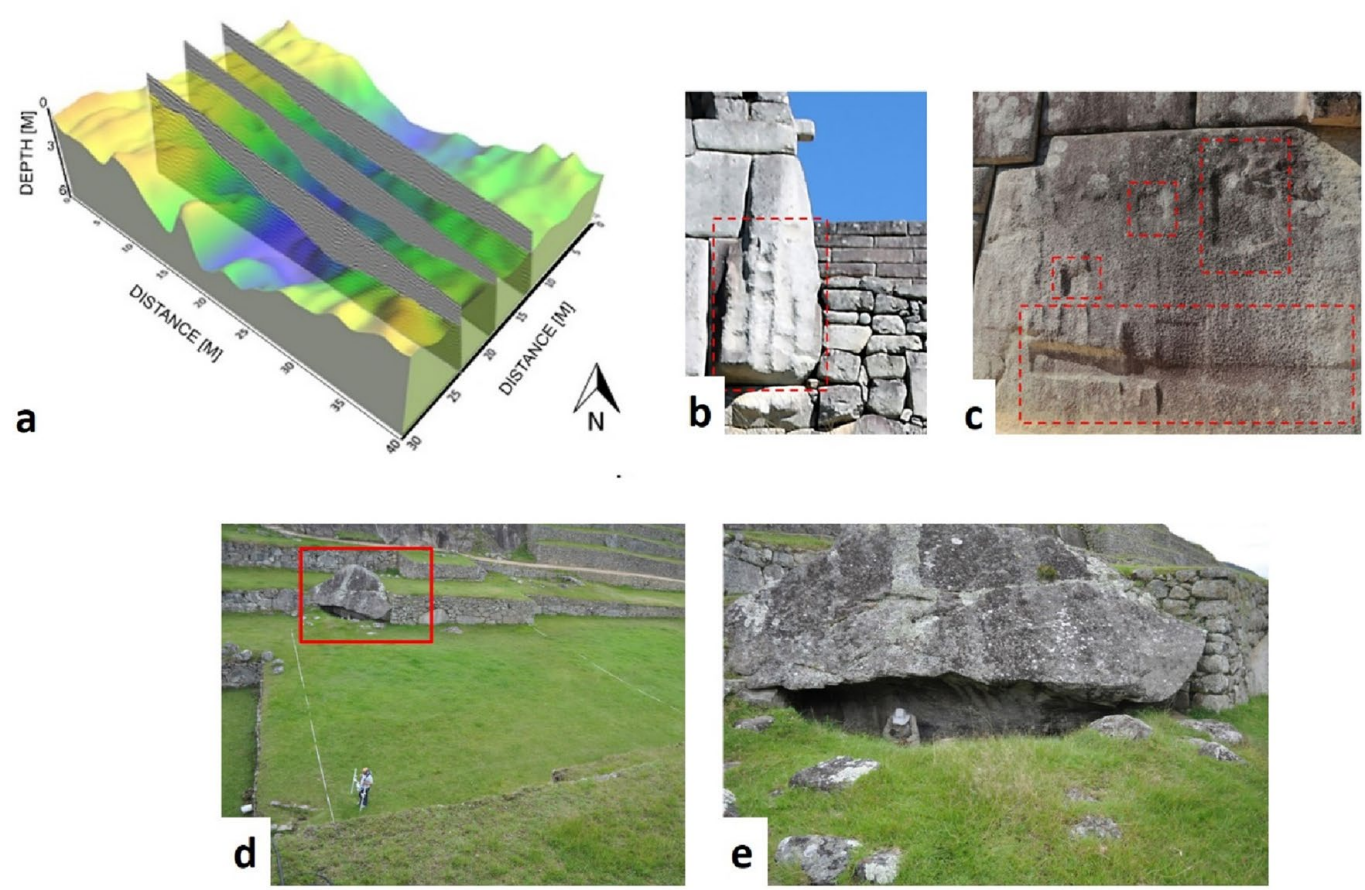
(**a**) Reconstruction of the jagged concave bedrock characterizing the *Plaza Principal* obtained with the picking of the GPR reflections imputable to the batholiths. (**b**,**c**) Signs of quarrying activity visible on granite blocks of the Main Temple (**b**), and Priest’s House (**c**) (photo by N. Abate). (**d**) *Plaza Principal*: red box indicates a rocky body of the bedrock emerging above the ground level of the Plaza; (**e**) zoomed detail of the rocky body seen in 7d (photo by N. Masini).

The surveys highlighted the presence of an impluvium (Fig. 3g), first identified by crop marks from the satellite and UAS imaging, and later confirmed by the geophysical prospections. The integration of results from diverse remote sensing technologies documented the existence of a watershed ("Phase 0: the drainage basin"), oriented in the EW direction (with a maximum depth of around 3–3.5 m). The integration of GPR and ERT imaging allowed the estimation of the granite bedrock depth and the characterization of its shape. The 3d model, generated by GPR (Figs. 5b, 8a), along with the georadar and ERT sections (Fig. 4a–d) confirmed the presence of a buried drainage basin along with the jagged surface of the bedrock resulting from the quarrying activities (see "Phase I: the quarry").

The signs of the ancient quarrying are still today visible on numerous large blocks as, for example, those set along the terrace wall overlooking the Plaza Principal and those close to the Main Temple and the Priest’s House in Sacred Plaza (in Fig. 8b,c). Moreover, several shaped big block stones emerge along with rocky bodies (part of the bedrock of the catchment area) fully integrated into the retaining structures of the *andenes* (Fig. 8d,e).

### The Plaza Principal as a work in progress: from natural catchment area to the Plaza in two phases

The EO-based results point out that, despite the well-thought-out architectural plan following a relative constructive coherence, the Plaza Principal of Machu Picchu was subsequently modified (see "Phase II: The Plaza"). The Plaza Principal had undergone several changes likely to accommodate larger public gatherings.

The GPR survey (E1, E2, E3) (see Fig. 7) (D1) revealed that the Plaza Principal was developed in two constructive phases as evident for the southeast area.

The first phase was related to the setting of a *plaza hundida*, namely a sunken plaza. This architectural feature was a common ritual space in the Inca sites, sometimes connected to ritual baths as in the case of Chachabamba^23,25^.

The second phase consisted of the extension and elevation of the Plaza Principal made to expand the area for ritual and social activities along the NW–SE axis, above the originally hydrographic basin.

As a whole, around the 60% of the construction efforts were needed to reshape the water catchment for the drainage system^6^. It is widely recognized that the Incas were masters in hydraulic engineering, particularly in water conveying and management systems^26^. The *llaqta* of Machu Picchu is undoubtedly an outstanding example of the Inca achievements in the design, construction, and management of surface and underground drainage systems. The system remains in use today and is effective in preventing water logging, soil erosion, and collapse of walls^6^. This stable foundation is the primary reason why the temples, buildings, and agricultural terracing systems remain standing even after centuries of abandonment and heavy rainfalls.

The architectural evidence has helped scholars to understand the efficiency of water runoff and drainage systems^6^. Less visible and understood are the subsurface infrastructure. The integration of the archaeological data with the results of geophysical and multispectral imaging advanced our knowledge and contributed to formulate some hypotheses on the diverse building phases.

Results from satellite, UAS, and geophysical surveys provided evidence of a buried drainage system which exploits the sloping soil layers and bedrock (detected by GPR) to direct the waters towards the southeast side of the Plaza Principal (Figs. 5d, 6c). This hypothesis is confirmed by the excavations that revealed the overlapping of the diverse stratigraphic levels characterized by different granulometry and consistency (sandy silt, silty sands, and silty sands with gravel), devised to increase the permeability (see "Introduction", and Figs. 1f, 2a).

The Incas used to exploit the natural capability of a basin to drain water, maintaining its effectiveness even in the dry season. This has been conceptualized and modeled by Fairley^27,28^ based on geologic water storage. The Incas used to manage an aquifer system building a wall across the former discharge boundary. This way the exiting water was forced to be stored close to the wall and then conveyed to a single drain. The water system was adequately controlled and channeled, and the water was leveraged for multipurpose functions^6,11,21,22,29^ including the ceremonial activities.

The capability to control the water flow was considered an evidence of the divine nature of the Incan Emperor. For this reason, numerous Inca hydraulic structures related to water management were conceived and realized as monumental or ceremonial architecture, as, for example, the exceptional water structures of Ollantaytambo^9,30^, Tambomachay^31^, Pisac^32^, Sacsayhuaman^33,34^ in the Cusco and *Valle Sagrada* area. Moreover, there are numerous well-known examples of the use of hydraulic architecture for ceremonial and, by extension, for political purposes in diverse sites, as in Tawantinsuyu, the Inca Caranqui^35^, and Ingapirca^36^, in Ecuador, Namachuco devoted to Apu Catequil^37^ or the most emblematic example in Saywite^38^.

Moreover, close to Machu Picchu, there are several sites with very developed water systems as, Chachabamba^23,25^, Choqesuysuy^39^, Phuypatamarca, Qantupata or Wiñay Wayna^40^.

**Figure 9:**
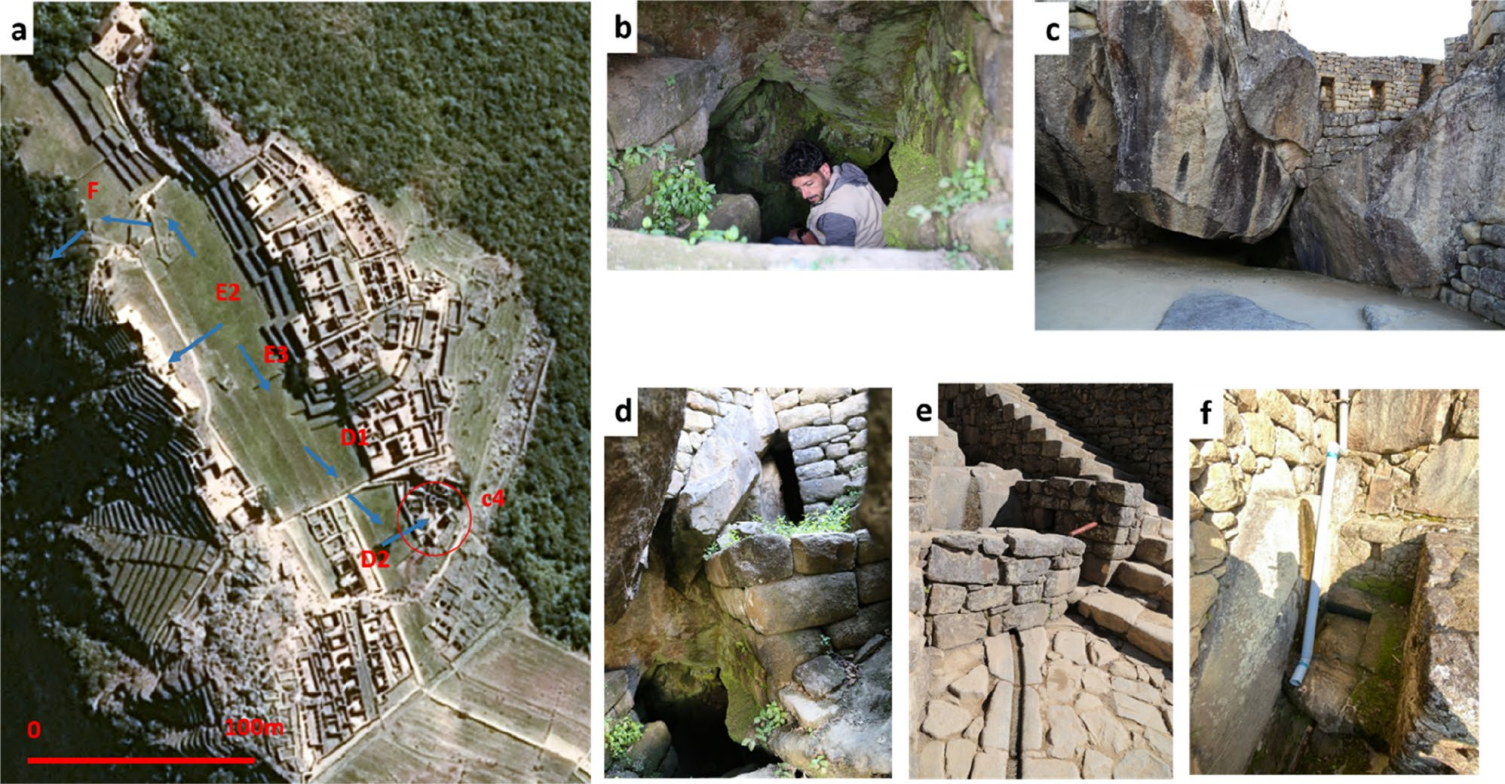
(**a**) Hypothesis on the underground water drainage system. Light blue arrows indicate the water flow direction; the red circle denotes the architectural complex of Temple of the Condor towards which part of the water drained south of the Plaza Principal is conveyed. (**b**–**f**) Detail of the Temple of the Condor. (**b**) and (**d**) show a tunnel which in the past conveyed the water towards the Tempe of Condor. (**e**) Detail of a canal and a rock of the ritual space of the temple. (**f**) Bath next to the Temple of the Condor.

In Machu Picchu, the drainage system of the Plaza Principal was made likely to drain the *andenes* system up to the north (Fig. 9a, F) and south (Fig. 9a, D1, and D2). This hypothesis was corroborated by archaeological evidence from the east side of Plaza D2, where a tunnel was built to transport the water to the Condor Temple (Fig. 9b–e), set over the contours of the rocks and characterized by large stone seen as the representation of a condor. The Temple of the Condor is a complex of buildings which include caves used for ceremonial activities. South of the Condor complex there are several buildings with privileged access to the water from sacred bath system as common in the Inca sacred areas. One of these unique baths is located right next to the Temple of the Condor (f) and likely in the past connected to the system of water draining.

**Figure 10:**
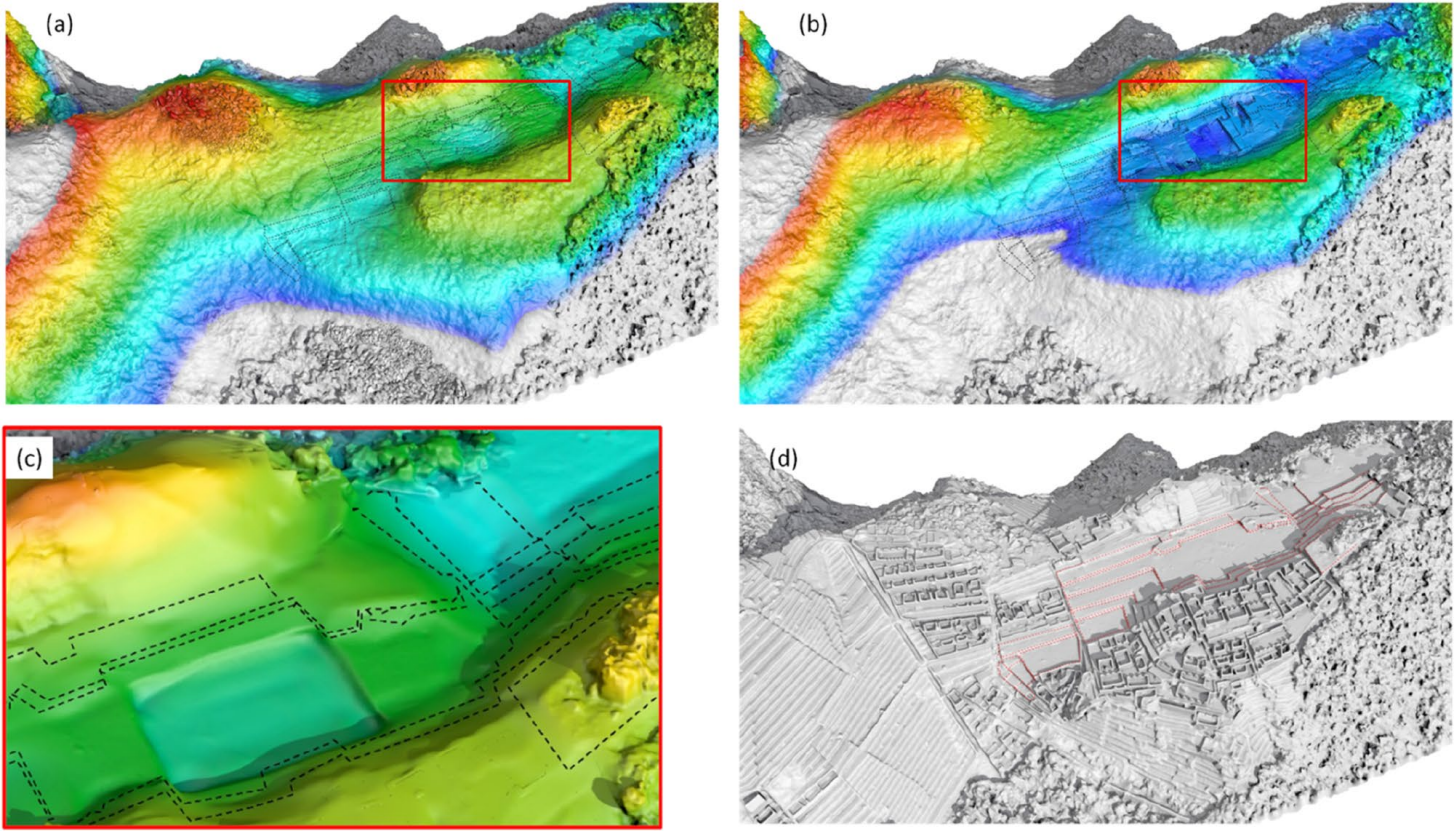
(**a**–**c**) Reconstructive hypothesis of *llaqta* of Machu Picchu during the preparation phases of the site: from the water catchment (**a**) to the quarry (**b**), up to the Plaza Principal, in turn built in two phases, the first relating to the *plaza hundida* (**c**). Finally, image (**d**) depicts the last configuration of the Plaza, and, all around, the *andenes*, the buildings, and the temples.

The water management system was developed in two constructive phases (see Fig. 10), as the Plaza Principal. It is worth to mention an important finding related to the construction of water systems in the urban sector. The Incas planned to replace the segment of the water supply channel located in the Urban Sector originally built with irregular stones joined with clay. The aim was to replace the old structure with around fifty new lithic elements that were found scattered in the 7th platform of the Agricultural and Urban Sectors. This modification would have prevented water infiltration that would affect the structures of the Temple of the Sun complex^27^. This clearly shows that the Inca were aware of the problems of water infiltration, and able to change designed plans to address unexpected issues.

Like many Andean cultures, the Incas understood and sought to control natural phenomena, such as water with innovative hydraulic and environmental engineering techniques. In addition to these practical considerations, the control of water was presented as political and sacred power (29).

## Conclusions

Machu Picchu with its associated sites has long puzzled scientists for many reasons, as, its location, the highly sophisticated Inca capability of adaptations, the hypothesis that it was never finished.

The herein devised non-invasive investigations enabled the reconstruction of the first building phases including the initial preparation one. Multiscale and multisensor EO techniques (including geophysics) documented the anthropogenic layering of the subsoil, thus allowing the recreation of initial pre-construction setting and unveiling the Machu Picchu environment before the construction that we know nowadays. This area first served as a quarry, subsequently reshaped and then secured through adequate drainage systems (see Fig. 10).

As a whole, the devised non-invasive analyses.

(i) enabled the identification and characterization of the diverse phases of the construction site;
(ii) revealed that the Plaza Principal was developed in two constructive phases, the first phase was related to the setting of a *plaza hundida*, namely a sunken plaza and the second phase related to both the extension and elevation of the Plaza Principal to expand the area for ritual and social activities;
(iii) unveiled the buried drainage systems adopted for the andenes as for the whole site to drain water and to prevent structural collapses. The drainage systems are still effective today, as evident by the fact that the abandoned site remained stable for centuries without maintenance;
(iv) improved our understanding of the Inca's capability to confront geomorphology and hydrogeological hazards with highly sophisticated and effective environmental engineering interventions fully integrated with nature and the sacred landscape, result of a local evolution of more ancient contruction cultures, including the Tiwanaku one^9,10,41^.

Some examples of the Incas achievements are evident in the drainage systems, still effective today, and in the like terraces (*andenes*) made as wide steps to stabilize the site, whose slopes exhibit debris accumulation as a result of past and present landslide activity^42^, and efficiently designed reshaping the gradient of the slopes for several functions: (a) for risk mitigation, protecting from uncontrolled runoff and hillside erosion, (b) for agricultural purposes to gain land for food production, and also (c) as a complementary part, for the most important ceremonial constructions. Incas were certainly the first experimenters and users of Nature Based solutions for risk mitigation purposes.

## Methods

This section explains the methodological approaches used for the purpose of our investigations and additional details are in the Supplementary Information (SI).

Results from non-invasive multisource prospections were coupled with archaeological records in order to identify and characterize the diverse phases of the site-transformation and arrange a relatively complete picture of the construction process. Findings from the archaeological excavations facilitated the interpretation of the results from EO surveys which provide a broad subsurface imaging of the Plaza principal (the original core of the whole archaeological area).

Five complementary survey methods were used to investigate the subsoil at different depths (see SI): multispectral imaging from (i) satellite and (ii) UAV to identify and map the presence of buried structures or pits and ditches through archaeological proxy indicators visible in the surface; (iii) electrical resistivity tomography (ERT) to characterize the electrical behavior of the subsoil up to 10 m; (iv) Ground Penetrating Radar (GPR) to detect and image objects, bodies and anthropogenic layers reflecting electromagnetic waves, up to an expected depth of 2 m; (v) magnetic surveys in a gradiometric mode to detect and map variations of the magnetic earth’s field referable to any anthropization processes.

Several advantages are expected using different survey methods, as: (i) overcoming the intrinsic limitation of a single method including effectiveness, time, and cost for the acquisition, (ii) performing investigations at diverse spatial scales, (iii) sensing the subsoil at different depths, thus facilitating the archaeological interpretation.

### EO based methodological approaches

#### Satellite and UAS data set

The Very High Resolution (VHR) satellite data set, used for the purpose of our analyses, was made up of multi-temporal, multi-sensor, multispectral images. The UAS survey was carried out employing a DJI Phantom 3 Professional, equipped with the owner RGB camera and with a Parrot Sequoia multispectral camera. The acquired images were radiometrically corrected thanks to the use of a Parrot Sequoia reflectance panel, captured before and after each flight. Finally, in order to work on a GIS basis with all data from remote sensing (drone and satellite) and geophysical data, several ground control points (GCPs) and ground validation points (GVPs) were surveyed with a high-precision GNSS. These points were then used (i) for the correction of the photogrammetric processes and (ii) for the correct georeferencing of the datasets (process described in SI and Fig. S3).

#### Satellite and UAS data processing

The data set, acquired from both satellite and UAS survey, was processed following the flowchart in Fig. S1 in SI, devised to extract information and make comparable the results related to the different spatial scales (0.3–0.5 m for satellite, and 0.04 m for UAS). For each year, the multi-band images were processed to compute spectral indices (formulas are listed in Table S1 in SI) to enhance archaeological features (see also^43–51^).

#### Electrical resistivity tomography

Electrical resistivity tomography (ERT), is a geophysical method based on the imaging of the electrical resistivity distribution within the subsoil by injecting a current into the ground and measuring the related potential drops^52^.

The ERT surveys were carried out using Dipole–Dipole (DD) and Wenner–Schlumberger (WS) acquisition schemes; the former for its ability in detecting lateral resistivity variations and the latter for its higher signal-to-noise ratio and for its sensitiveness to vertical discontinuities^53^. DD and WS data were collected in both direct and reverse mode. This last mode is based on the “reciprocity principle”^54^ and consists in inverting the position of the current and potential electrodes.

The geoelectrical data were inverted using the commercial software RES2DInv. A synoptic view of ERT results is in Fig. S7 (for additional detail see Supplementary Information and^55,56^).

#### GPR investigations

The GPR exploits radar pulses to image the subsurface and using antennas with different operating frequencies, the method permits an adequate resolution and depth of investigation for the most common archaeological applications^57–59^.

The GPR data were acquired using the system TH Dual-F Hi-Mod (IDS), equipped with a multi-frequency (200 and 600 MHz) antenna. The presence of obstacles, as megaliths, stones, irrigation pipes was considered for supporting the interpretation.

Raw data were processed using the following processing chain (shown in Fig. S8 in SI):

- Time gating for removing the reflections due to the air layer between the antenna and the subsoil surface; in this way, direct waves effects were deleted.
- Background removal to remove the background noise. To this purpose an average trace is calculated for the entire radargram and then subtracted to every single GPR trace, sample by sample.
- Signal gaining with ACG filter to provide a time-varying enhancement of signal amplitudes. The filter performs a subtraction between the average amplitude of a signal in a well-known time-window and the maximum amplitude of the overall trace. To this aim the time window chosen was equal to 70 ns and 30 ns for the 200 MHz and 600 MHz data, respectively.
- Band-pass filtering to remove the noise due to non-coherent loss of the signal able to limit the signal to noise ratio and the surrounding media. The filter works within the frequency domain and acts on each trace independently. For the data acquired at the nominal frequency of 200 MHz, only the signal included between 75 and 350 MHz was considered.
- Kirchhoff-migration for the time-depth conversion, performed after the evaluation of the characteristics of the subsoil. To this aim the velocity estimated was equal to 0.07 mn s^−1^.
- Normalization of the amplitude (performed on the mean amplitude value of the complete profile) to de-clip saturated traces using a polynomial interpolation (for additional details see SI and^60-64^).

#### Geomagnetic prospections

The geomagnetic method (MAG) is based on the mapping of local variations of the Earth magnetic field resulting from changes of magnetic properties of the underlying rocks or from the presence of buried artifacts within the subsoil^65,66^.

The MAG data were acquired in gridded areas of various sizes (ranging from 20 × 20 m to 40 × 40 m) using survey procedure that are standards in archaeological prospection. Calibration was performed on-site prior to the acquisition through an automated procedure which corrects possible misalignment in the sensor measurements.

Standard processing procedures, using signal and image processing techniques, were applied and the magnetic data were rendered as an image. Vertical gradient maps were produced applying a minimum curve interpolation (“spline”) to smooth.

### Archaeological records

In 2016 and 2017 two excavation units were performed^14,19,24^, in the areas shown in Fig. 1f and labeled as UE25 (11.70 × 5 m) and UE13 (6 × 6 m) (see also Fig. 2a,b). These excavations revealed layers of different soil types and colors representing two distinct construction phases: 1. the stabilization of the lower platform by filling and setting water drainage systems, and: 2. sculpt the present stepped (see additional information see Graphical summary in Figs. S13–S15 in SI).

The two excavation units presented even limited but significant findings related to the construction phases. To extrapolate these results across the entire Plaza, non-invasive Earth Observation (EO) surveys were conducted (for additional detail on EO data integration see^67-73^).

### Supplementary Information

Supplementary Information.

## Data Availability

The datasets used and analysed during the current study are available from the corresponding author on reasonable request.
