# Author Correction: Non invasive subsurface imaging to investigate the site evolution of Machu Picchu

**DOI:** 10.1038/s41598-023-46706-8

**Published:** 2023-11-07

**Authors:** Nicola Masini, Gerardo Romano, Dominika Sieczkowska, Luigi Capozzoli, Daniele Spizzichino, Francesco Gabellone, Jose Bastante, Manuela Scavone, Maria Sileo, Nicodemo Abate, Claudio Margottini, Rosa Lasaponara

**Affiliations:** 1 CNR-Institute of Heritage Science, C.da S. Loya, 85050 Tito Scalo, Italy; 2 University of Bari, Bari, Italy; 3 Silesian University of Technology, Gliwice, Poland; 4 CNR-Institute of Methodologies for Environmental Analysis, C.da S. Loya, 85050 Tito Scalo, Italy; 5 ISPRA, Geological Survey of Italy, Rome, Italy; 6 CNR, Nanotech, Lecce, Italy; 7 Peruvian Ministry of Culture-Directorate of Culture of Cusco, Programa de Investigaciones Arqueologicas e Interdisciplinarias en el Santuario Historico de Machupicchu (PIAISHM), Cusco, Peru; 8 UNESCO Chair at Florence University, Florence, Italy

Correction to: *Scientific Reports* https://doi.org/10.1038/s41598-023-43361-x, published online 25 September 2023

In the original version of this Article Dominika Sieczkowska was incorrectly affiliated with ‘Centre for Andean Studies, University of Warsaw, Warsaw, Poland’. Additionally, Nicodemo Abate was incorrectly affiliated with ‘Silesian University of Technology, Gliwice, Poland’, as well as ‘Centre for Andean Studies, University of Warsaw, Warsaw, Poland.’ Their correct affiliations are listed below.

Dominika Sieczkowska:

Silesian University of Technology, Gliwice, Poland.

Nicodemo Abate:

CNR-Institute of Heritage Science, C.da S. Loya, 85050, Tito Scalo, Italy.

The original Article and accompanying Supplementary Information file have been corrected.

