# Supplementary material for: Non invasive subsurface imaging to investigate the site evolution of Machu Picchu
Source: Sci Rep. 2023 Sep 25;13:16035. doi: 10.1038/s41598-023-43361-x (PMC10519973; doi:10.1038/s41598-023-43361-x)
Supplement: Supplementary file 1 — Supplementary Information. [file 41598_2023_43361_MOESM1_ESM.docx]

**APPENDIX**

**Supplementary Material for**

**Non invasive subsurface imaging to investigate the Site evolution of Machu Picchu**

Nicola Masini^(1)^, Gerardo Romano^(2)^, Dominika Sieczkowska^(3)^, Luigi Capozzoli^(4)^, Daniele Spizzichino^(5)^, Francesco Gabellone ^(6)^, Jose Bastante^(7)^, Manuela Scavone^(1)^, Maria Sileo^(1)^, Nicodemo Abate^(1)^, Claudio Margottini^(8)^, Rosa Lasaponara^(4)^ .

**(1) CNR - Institute of Heritage Science, C.da S. Loya, 85050 Tito Scalo, Italy, nicola.masini@cnr.it**

**(2) University of Bari, Italy**

**(3) Silesian University of Technology, Gliwice , Poland**

**(4) CNR - Institute of Methodologies for Environmental Analysis, C.da S. Loya, 85050 Tito Scalo, Italy**

**(5) ISPRA, Geological Survey of Italy, Rome, Italy**

**(6) CNR, Nanotech, Lecce, Italy**

**(7) Peruvian Ministry of Culture - Directorate of Culture of Cusco, Programa de Investigaciones Arqueologicas e Interdisciplinarias en el Santuario Historico de Machupicchu (PIAISHM), Peru**

**(8) UNESCO Chair at Florence University**

**The Appendix includes: Extended Methods and Methods (in sections A, B, C, D), Figures S1 to S15, Table S1**

**Extended Methods and Materials**

**A - Multispectral remote sensing analysis**

The data set, acquired from both satellite and UAS survey, was processed following the flowchart in Figure S1, devised to extract information and make comparable the results related to the different spatial scales (0.3-0.5 m for satellite, and 0.04 m for UAS).


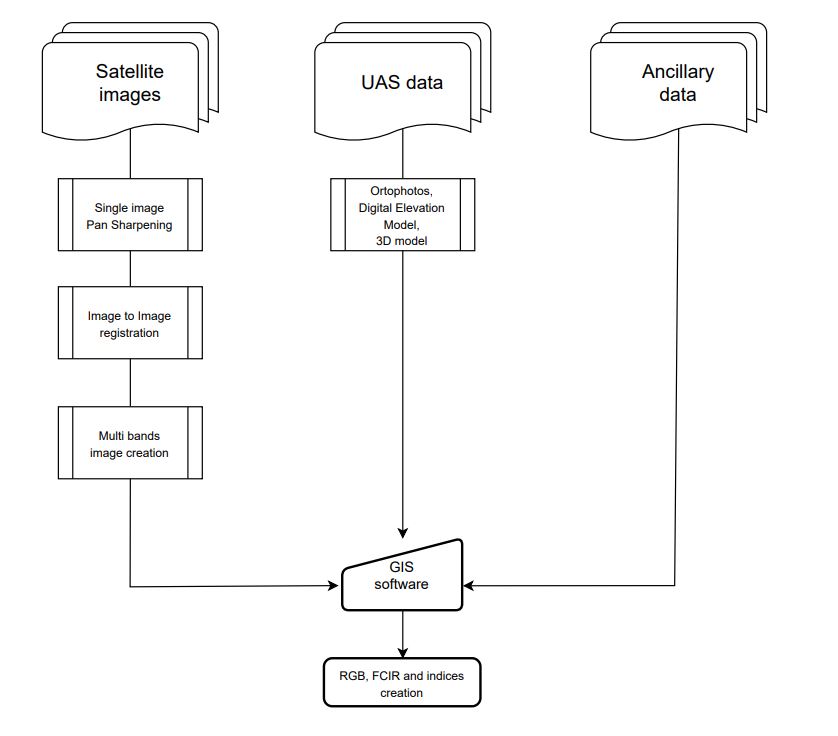


Figure S1 -Flowchart of remote sensing data processing of multispectral satellite and UAS imagery

**A.1 Satellite data and processing**

The satellite data set was made up of multi-temporal, multi-sensor, multispectral images, captured by GeoEye-1 (August 6^th^, 2016, and June 19^th^, 2019), WorldView-3 (July 18^th^, 2018).

The high-resolution offered by WorldView (MS: 1.2 m, Pan: 0.3 m) and GeoEye (MS: 2m, Pan: 0.5m) satellites have been used for years in the analysis of archaeological areas and the discovery of new monuments with great results [51-52].

These satellites provide multispectral images in Blue, Green, Red, and Near Infrared bands, and a very high resolution panchromatic band herein merged using the SPEAR Pan Sharpening function present into the ENVI software tools.

To enhance archaeological features, pan-sharpened images were combined using both RGB (R: Red band; G: Green band; B: Blue band) and False Colour Infrared Composite (R: Near Infrared band; G: Red band; B: Green band) (Figure S2).

The multi-band images (B1: Blue, B2: Green, B3: Red, B4: Near Infrared) for each year (2016, 2018, 2019) were further processed to compute spectral indices (mathematical combination of diverse spectral bands) to enhance archaeological features.


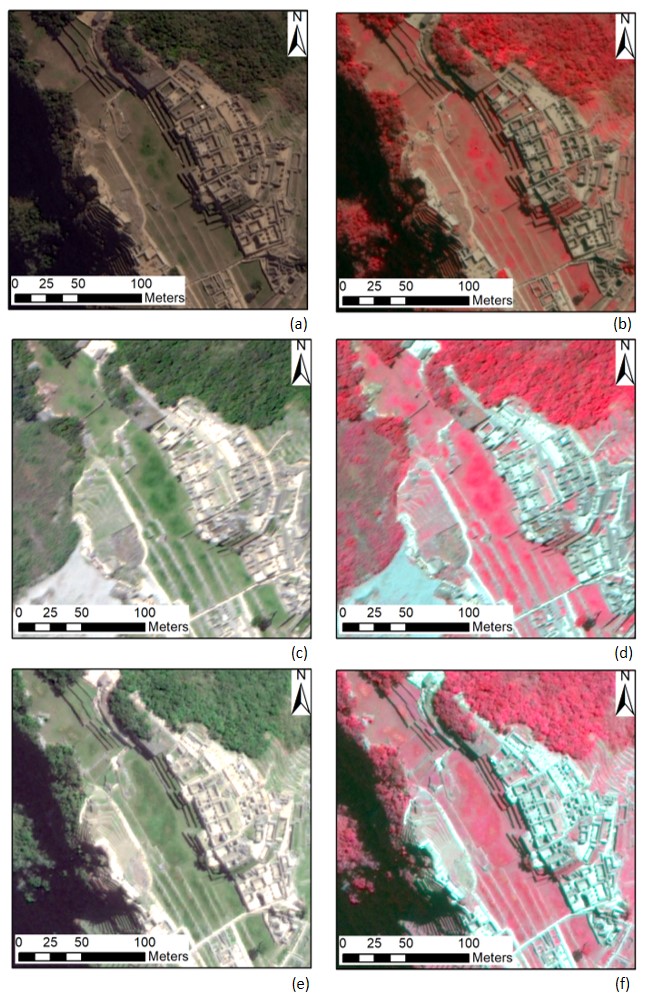


Figure S2 RGB_FCIR – Satellites images: (a) 16 Aug 2016 (RGB); (b) 16 Aug 2016 (FCIR); (c) 18 Jul 2018 (RGB); (d) 18 Jul 2018 (FCIR); (e) 19 Jun 2019 (RGB); (f) 19 Jun 2019 (FCIR).

**A.2 UAS based data acquisition and processing**

UAS investigation in Machu Picchu was carried out by a DJI Phantom 3 Professional (equipped with the owner RGB camera) and a Parrot Sequoia multispectral camera.

Phantom 3 Professional has a 12mpx global shutter camera, while the Parrot Sequoia camera is composed of: (i) a module with four single-band 1.2 mpx cameras (green, red, red-edge, near-infrared) and an RGB camera, (ii) a sunlight sensor useful to capture the lighting conditions at the time of shooting.

The acquisition phase was carried out from 4^th^ to 6^th^ of June 2019, between 6:20 and 6:50 (local time).

The choice of the time of acquisition was strongly influenced by the touristic flux as well as by the sunset time (17:50 local time).

The survey covered the area of the Plaza Principal and the nearby terraces.

The flight was conducted automatically after creating a flight plan thanks to the Pix4D application.

The acquired area was 0,040 km2 (4,0366 ha). During each session, the drone reached a height of 40 m above the take-off point and a cruising speed of 2 m/s (7.2 km/h). The photos overleap was constant, with a frontal and lateral overlap of 85%.

The Sequoia camera, which is not directly connected to the Pix4Dcapture application, has been set to Time-laps mode, with a shutter speed of 1.75 sec.

In this way, it was possible to capture RGB images at 12Mpx, thanks to the camera integrated in the Phantom 3 Professional, and multispectral images at the same time.

The acquired images were radiometrically corrected thanks to the use of a Parrot Sequoia reflectance panel, captured before and after each flight.

284 photographs were taken by DJI Phantom 3 Professional and 1952 by Parrot Sequoia camera, 488 for each channel (Red, Green, Nir, Red-edge), and data were processed using: (i) Pix4Dmapper Pro software (version 4.4.12), and (ii) Agisoft Metashape, for the creation of a three-dimensional model, DEM (Digital Elevation Model), reflectance maps, and orthophotomosaic, as shown in figure S3.

Several ground control points (GCPs) and ground validation points (GVPs) were surveyed with a high-precision GNSS. The acquired points were chosen according to a visibility criterion: (i) some high-visibility markers (29.7 x 42 cm with a circle target), (ii) circular landing pads (50 cm diameter), (iii) the basal part (i.e. edges and corners) of structures in the square and terraces, (iv) particularly prominent features. This operation was necessary in order to (i) anchor the three-dimensional RGB model to the three-dimensional multispectral model and (ii) georeference the geophysical data, which, having covered all the flat surfaces of the square and terraces, were georeferenced on the DEM (Digital Elevation Model) extracted from the photogrammetric process. In total, 28 GCPs and 11 GVPs were used. The software's estimated error on GVP positioning, after optimisation of the points, was ± 1 cm on X and Y axis and ± 2-3 cm on Z. The reference system used (SR) was WGS 84 - UTM 18S.


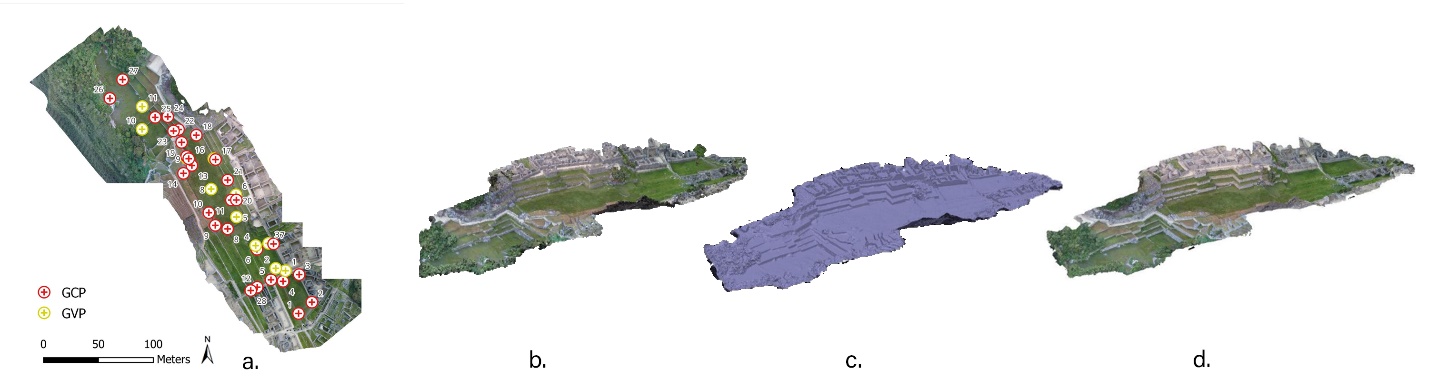


Figure S3 – (a) Position of Ground Control (GCP) and Ground Validation (GVP) points; (b) Dense Point Cloud; (c) 3D mesh; (d) Textured 3D model.

The produced ortophotomosaics (4.89 cm/pixel Ground Sample Distance), reflectance maps, and DEM were processed into the ArcMap GIS software, to compute the different spectral indices and enhance archaeological features [53].

**A.3 Spectral indices of multispectral satellite and UAS data.**

The spectral indices (see Table S1) considered for the purpose of our analyses were calculated according to the formulas listed in Table I for both multi-spectral satellite (paragraph 5.1.1) and *UAS* images (paragraph 5.1.2).

**Table S1.** Spectral Indices

| Index | Equation | Reference |
| --- | --- | --- |
| Difference Vegetation Index (DVI) | $NIR-\mathrm{Re}d$ | (54) |
| Green Difference Vegetation Index (GDVI) | $NIR-Green$ | (55) |
| Green Normalized Difference Vegetation Index (GNDVI) | $\frac{(NIR- Green )}{(NIR+Green)}$ | (55) |
| Green Ratio Vegetation Index (GRVI) | $\frac{NIR}{Green}$ | (56) |
| Normalized Difference VegetationIndex (NDVI) | $\frac{(NIR- \mathrm{Re}d )}{(NIR+\mathrm{Re}d)}$ | (57) |
| Optimized Soil Adjusted Vegetation Index (OSAVI) | $\frac{1.5\times(NIR-\mathrm{Re}d)}{NIR+\mathrm{Re}d+0.16}$ | (58) |
| Simple Ratio Nir/Red | $\frac{NIR}{\mathrm{Re}d}$ | (58) |
| Simple Ratio Red/Green | $\frac{\mathrm{Re}d}{Green}$ | (58) |
| Nonlinear Vegetation Index (NLI) | $\frac{NIR^{2}-\mathrm{Re}d}{NIR^{2}+\mathrm{Re}d}$ | (59) |

The maps obtained from the spectral index are shown in Figure S4.


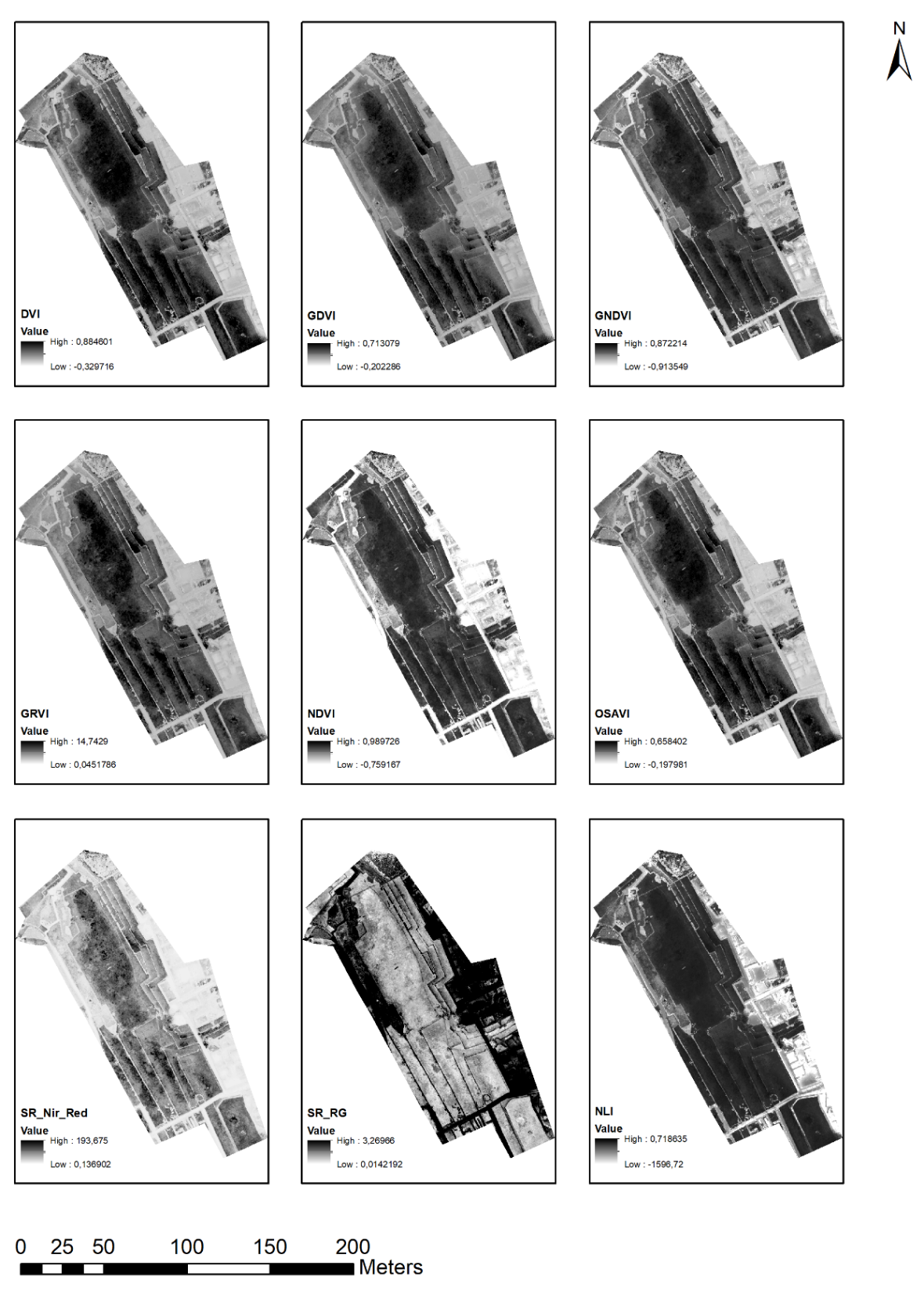


Figure S4 –Spectral index Maps obtained from the UAS multispectral images.

These maps enhanced some archaeological features located both in the central area or *Plaza Principal* and close to the *andenes*. In particular, for the Plaza Principal groups of features or proxy indicators are clearly enhanced, as below detailed:

1. large and medium crop-marks are clearly defined in the center and on the sides of the Plaza. The main central feature (40x22m ca) is linked with the trend of the underlying bedrock and well fit with the results from the geophysical data as shown in Figure S5;
2. traces of previous excavation or reconstruction of the Plaza (Figure S5),
3. traces of activity in the place, below the ground, produced by the use of tools or holes (Figure S5).


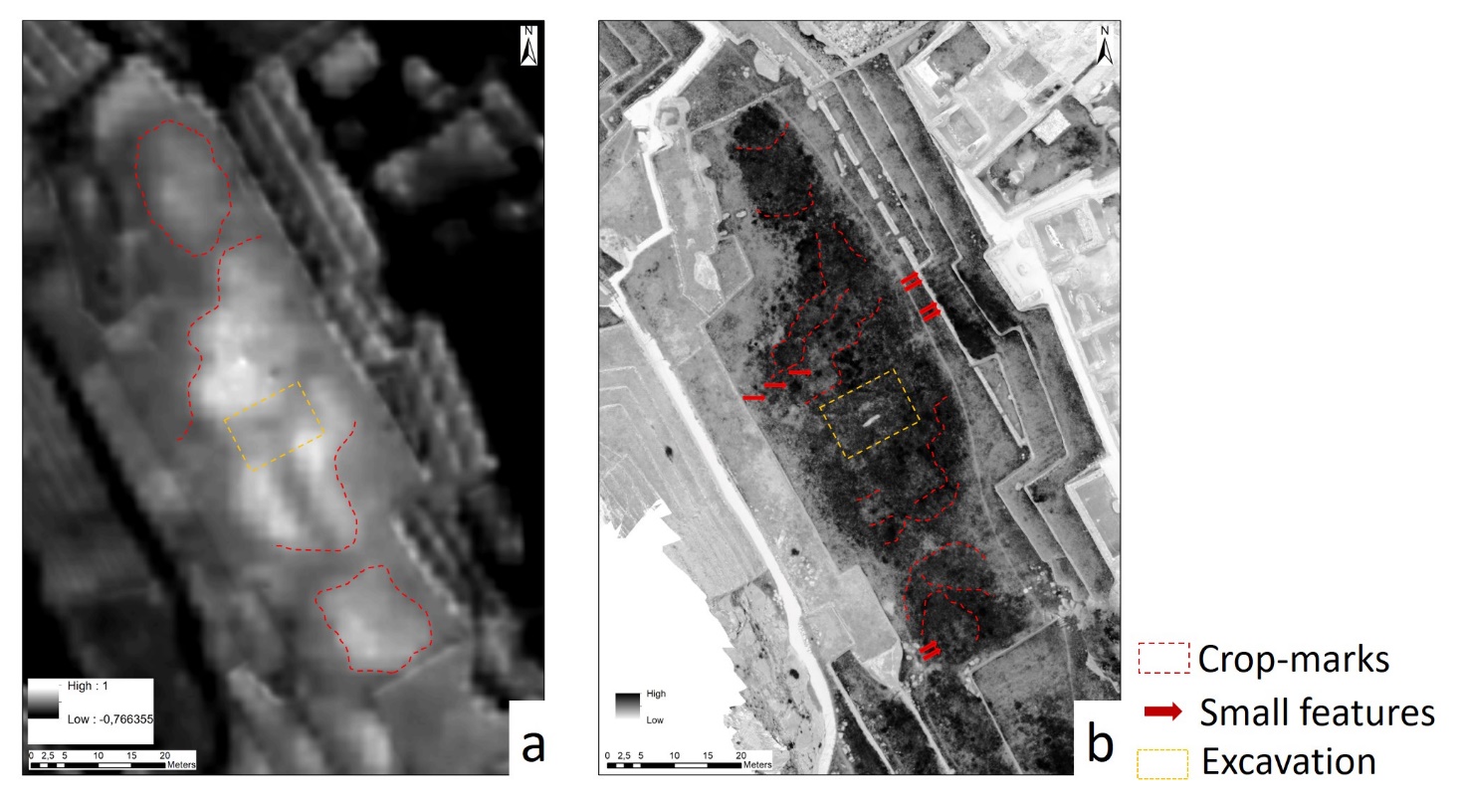


Figure S5 Crop Marks from: (a) World View-2 satellite NDVI map (16 August 2016), and (b) UAS based NDVI (4-6 June 2019).

The analysis of the crop-marks highlighted by satellite and UAS showed a complex framework already exposed in the sections of the paper (section 2). Some considerations can be made about the differences and similarities in the data collected by satellite and UAS. Both satellite and UAS images showed the large central crop-mark. However, the satellite showed a much higher enhancement than the UAS images for this large feature, although they are at a lower resolution, allowing us to appreciate the contours of the large crop-mark probably created during the rearrangement of the basin (Phase 0; showed in section 2.1), to square (Phase II in section 2.3). This phenomenon seems to be produced by two factors mainly related to the spatial resolution and the time of the image acquisition. It is known that crop-marks visibility increases only in some periods of the year, in certain years, and under certain conditions. It is possible to hypothesize that, in this case, the visibility is increased mainly by the lower resolution, compared to the noisier UAS images, due to both the diverse condition of the surface when the images were sensed and also to some signal interpolation phenomena, which benefit the strong and persistent signal of the lush vegetation in the crop-mark, reducing those produced by noise elements. However, the UAS imagery allowed the discrimination of small-features probably produced by the anthropogenic action on the bedrock, following the operations that took place in the Plaza starting from the quarrying phase (Phase I in section 2.2).

The indices produced by UAS multispectral imagery enhanced small depth variations in the subsoil, which are evident in the state of vegetation/soil on the surface, as in the features identified in Plaza D, south of *Plaza Principal* (Figure 6). Probably, the visible features, according to geophysics and ancillary data, are the result of a rearrangement of the area following the different phases of the Plaza's expansion and rearrangement of the neighbouring area, by filling steps to create flat ground and arrange new drainage paths (Phase II) (Fig. 8; Fig. S4).


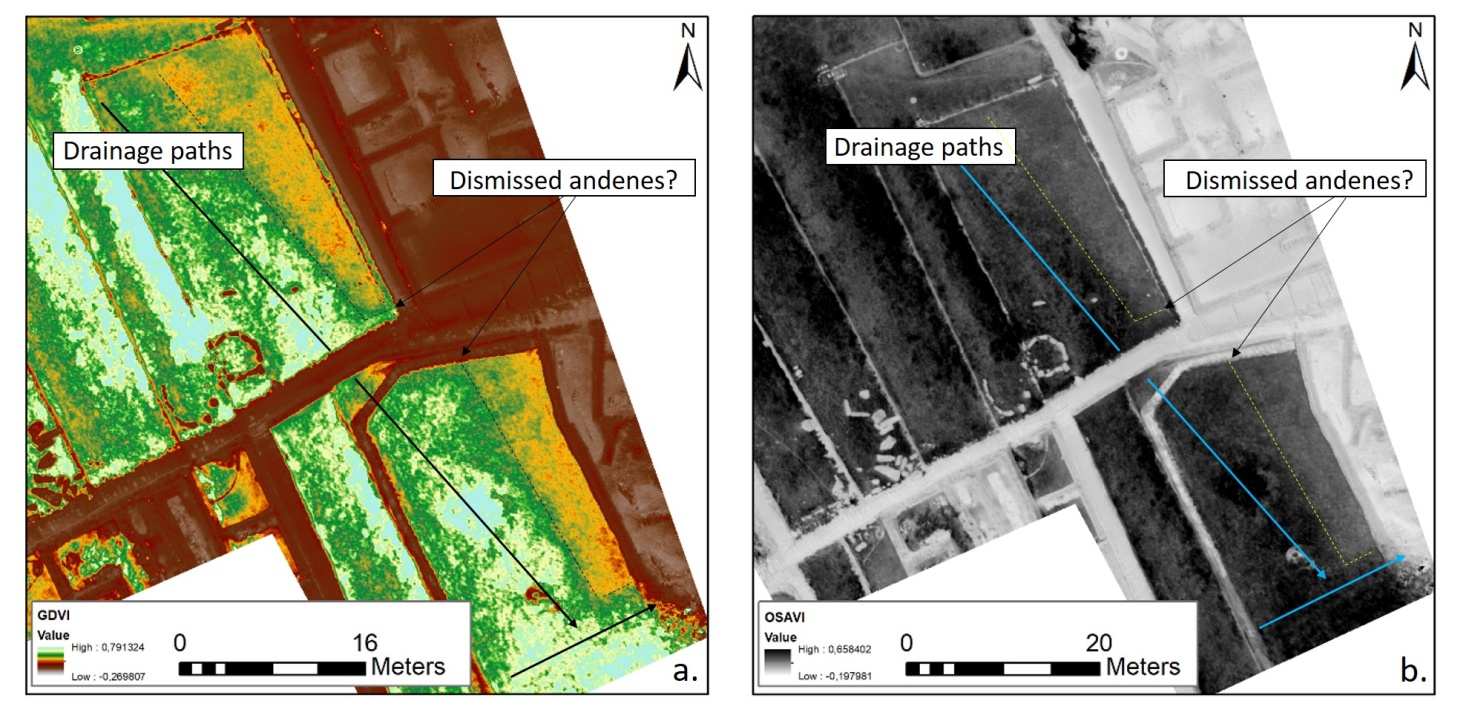


Figure S6 – UAS indices for site reconstruction and identification of archaeological proxy indicators: a. GDVI, b. OSAVI.

**B Geophysical based in Machu Picchu**

**B.1 Electrical Resistivity Tomography: method description, data acquisition, processing, and results**

Electrical Resistivity Tomography, also known as ERT, is a geophysical method able to image the electrical resistivity distribution within the subsoil by injecting a current into the ground and by measuring the related potential drops. .

All the ERT surveys in the areas of the monumental site of Machu Picchu were acquired with the aim to maximize the possibility to gather geological and archaeological information. Thus Dipole-Dipole (DD) and Wenner-Schlumberger (WS) acquisition schemes were used; the former for its ability in detecting lateral resistivity variations and the latter for its higher signal-to-noise ratio and for its sensitiveness to vertical discontinuities [60]. DD and WS data were collected in both direct and reverse mode. This last mode is based on the “reciprocity principle” [61] and consists in inverting the position of the current and potential electrodes. The comparison and the integration of reverse mode data with the direct mode data helps the geophysicist in asses the error level associated to its dataset and to improve the quality of the ERT results. The data collection with both DD and WS scheme allows also a creation of a “mixed” dataset or a “non-conventional” dataset whose inversion [60] may provide better results, in terms of resolution, compared to those that can be obtained by inverting DD and WS dataset separately.

In sector E2 of the *Plaza Principal* (for its location see Figure 1e), ERT data were collected along 32-meter-long profiles, spaced 1.5 meter apart, and by using an electrode spacing of 0.5 m. To cover each profile length, the roll-along technique was applied. Data were firstly acquired by using 48 electrodes covering 23.5 m and then the position of eighteen electrodes was shifted to complete each profile.

DD data (direct and reverse) were collected along all the profiles. Every six lines, starting from line 1, WS data (direct and reverse) were collected too. The choice to not acquire WS data along each line as well as the spacing between the lines (1.5 meters) was connected only to the lack of time necessary to acquire a denser grid.

All the geoelectrical data have been inverted by using the commercial software RES2DInv. The differences in inversion results linked to the use of direct and reverse data is minimal and testify the general good quality of the ERT data.

A synoptic view of all the geoelectrical investigation results is presented in figure S7. The figure shows: at left, all the ERT profiles; on the bottom a zoom of the ERT profile passing nearby the *wanka*, and at right the resistivity distributions at different depth obtained by the interpolation of all the ERT profiles.


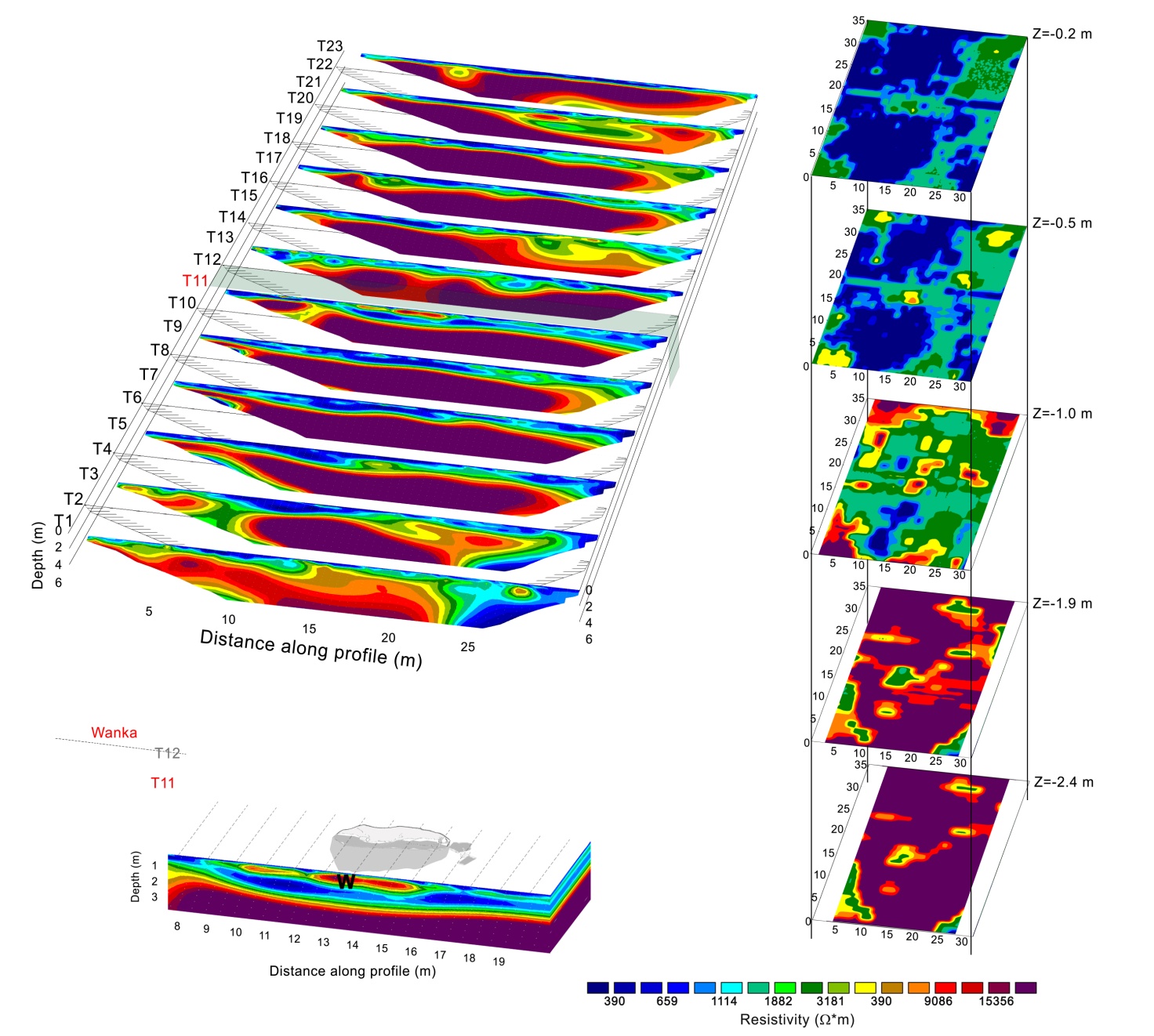


Figure S7 – (Left) Results of the ERT survey performed in E3 (for their readability only half of the acquired tomographies, only the odd ones, is shown in the figure). (Bottom) zoom of the ERT T11 crossing the wanka. (Right) resistivity slices at depths 0.2m, 1.0m, and 1.9m

In Figure S7 (left) it is possible to notice how all the ERT profiles show the same main features which are: a relatively more conductive layer (resistivity values less than 1000 Ω*m) with a mean thickness of about 50 cm overlying a high resistive electrostratum (resistivity values higher than 5000 Ω*m) possibly associated to the batholitic bedrock. The bedrock depth varies both along profile traces and from profile to profile. As shown for example along T1, T2, T15 and T21, the bedrock is characterized by discontinuities or large depression filled by a less resistive material (resistivity values ranging between 1000 Ω*m and 5000 Ω*m).

An association between resistivity values and geological formations can be done by examining resistivity distribution along profile T11 (panel b) where the results of archaeological excavation [23] can provide a robust constrain for the interpretation of the geoelectrical imaging. Accordingly to the archaeological excavation results and from field observations, the first layer (ρ < 1000 Ω*m) is mainly associated to the soft soil covering the sector E; the intermediate layer (1000 Ω*m <ρ< 5000 Ω*m) is composed of soil and of what is called “granitic chaos”, a mix of batholitic fragments of different size linked to the weathering of the batholitic rocks; finally, the third layer, the more resistive one, is associated to the bedrock, block of granitic batholits with a low fracturation level. According to this classification, it is expected that the resistivity of the first two layer may strongly vary as function of the soil water content while the same should be not true for the bedrock. The above proposed classification also explains the presence of the resistivity anomalies which can be observed at about 0.5m of depth (marked with the capital letter “W” in fig 16, bottom) and which are associated to the presence of the granite monolith (wanka).

To visualize the spatial distribution of the three identified electrostrata, a 3D reconstruction of the ERT results has been created. To this aim, a 3D interpolation of the ERT data was performed (figure S7, right). The colour scale adopted is the same of the ERT profile in which blue tonalities are mainly associated to the topsoil cover, green tonalities and yellow to the granitic chaos and red-violet ones the batholitic bedrock. In the first depth slice (Z=-0.2m), the topsoil cover is clearly present in the left half of the slice (from 0 to 20 m along x, and from 0 to 35m along y). Higher resistivity values are present in the upper right corner indicating the presence of granitic chaos. Going deeper (depth slice at Z=-1.0 m) a circular area (elliptical in the figure due to the distortion induced by the visualization projection) filled by granitic chaos is visible. The borders of this area, opened on its right side, are marked by the presence of the high resistive bedrock. Finally, the bedrock dominates the last depth slice (Z=-1.9 m). As it possible to see, the bedrock is interrupted in some isolated areas by the presence of the granitic chaos. It derives that, despite the relatively small extension of the investigated area, the bedrock is not imaged by the geoelectric method as a uniform regular surface.

**B2. GPR investigations: method description, data acquisition, processing, and results**

GPR exploits the reflections occurring in the subsoil due to the impedance electromagnetic contrasts occurring in the subsoil. Thanks to the possibilities to use antennas with different operating frequencies, the method permits an adequate resolution and depth of investigation for the most common archaeological applications [62]. Specifically, GPR allows the detection, localization, and reconstruction of sub-superficial target hidden in the subsoil analysing the electromagnetic field backscattered by the target when they are illuminated by an incident field [63]. Moreover, in favourable geological conditions as in the site of Machu Picchu, where occurs dry and low clay-content soils overlapped to batholiths, its use is also reliable for paleo-morphological reconstructions [64]. However, for supporting and validating the interpretation of the GPR data is strongly required its integration or comparison with direct data and results incoming from other remote sensing techniques, such as magnetometry, electrical resistivity tomographies or satellite image [65].

GPR data in Plaza Principal were acquired with the system TH Dual-F Hi-Mod (IDS), equipped with a multi-frequency antenna able to operate to the frequencies of 200 and 600 MHz. Data are collected in continuous and reflection mode with a time window of 130 ns (200 MHz) and 60 ns (600 MHz), samples per scan set at 512 with a resolution of 16 bits and a transmit rate of 100 kHz. The investigated area is flat and characterized by optimal conditions for the GPR data acquisition. Radargrams are acquired in time-mode using markers fixed every meter. The surveys are collected according to a regular grid that allowed to record radargrams every meter in two directions, mutually. The presence of obstacles, as megaliths or stones, presents in the area as well as irrigation pipes are accurately recognised and considered for supporting the interpretation of the data. The data were acquired in snake mode to reduce the time of acquisition. The prospecting is carried out with the help of measuring tapes and GPS station for accurately mapping the areas and georeferencing the geophysical data.

Raw data are processed with the software Reflex-W [66] to improve the signal noise ratio and to detect the most interesting features for archaeological and geological issues. The processing chain adopted is based on several step, as showed in figure S8:

1. Time gating for removing the reflections due to the air layer between the antenna and the subsoil surface; in this way, direct waves effects are deleted.
2. Background removal to remove the background noise. To this purpose an average trace is calculated for the entire radargram and then subtracted to every single GPR trace, sample by sample.
3. Signal gaining with ACG filter to provide a time-varying enhancement of signal amplitudes. In particular, the filter performs a subtraction between the average amplitude of a signal in a well-known time-window and the maximum amplitude of the overall trace. To this aim the time window chosen was equal to 70 ns and 30 ns for the 200 MHz and 600 MHz data, respectively.
4. Band-pass filtering of the data to remove the noise due to non-coherent loss of the signal able to limit the signal to noise ratio and the surrounding media. The filter works within the frequency domain and acts on each trace independently. For the data acquired at the nominal frequency of 200 MHz, only the signal included between 75 and 350 MHz was considered.
5. Kirchhoff-migration which enables the time-depth conversion, performed after the evaluation of the characteristics of the subsoil and the previous experiences made in areas with similar geological and archaeological conditions. To this aim the velocity estimated was equal to 0.07 mns^-1^.
6. Normalization of the amplitude (performed on the mean amplitude value of the complete profile) in order to de-clip saturated traces using a polynomial interpolation procedure.


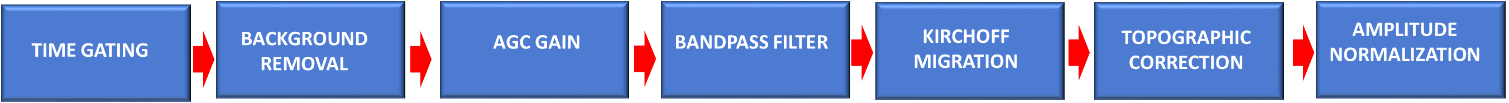


Figure S8- FLOW CHART of GPR data processing.

The high resolution obtained by the GPR acquisition allowed us to reconstruct the ancient bedrock. For this reason, a customised procedure, showed in figure S9, was used to highlight the most interesting anomalies present in the GPR data. The first operation consisted on the evaluation of the dielectric permittivity of the subsoil ε_R_ that enables us to convert the processed data from the time to the space domain. Further, by comparing the GPR data with the ERTs, and supported by the analyses of the hyperbolas generated by punctual elements present in the subsoil, a $\varepsilon_{r}$ value equal to 18 was estimated for an expected velocity of propagation of 0.07m/ns, approximately. This value characterizes the shallower anthropogenic level of subsoil, which corresponds to the presence of humid soil with intermediate water content. Assuming a homogenous velocity model of the subsoil for the level placed on the bedrock, the GPR data are converted from the time domain to the spatial domain assigning the real depths to the reflections due to the bedrock. The excavations carried out in the Plaza Principal (UE25 and UE13; see Fig. 1) gave us the possibility to validate the interpretation of the geophysical data.


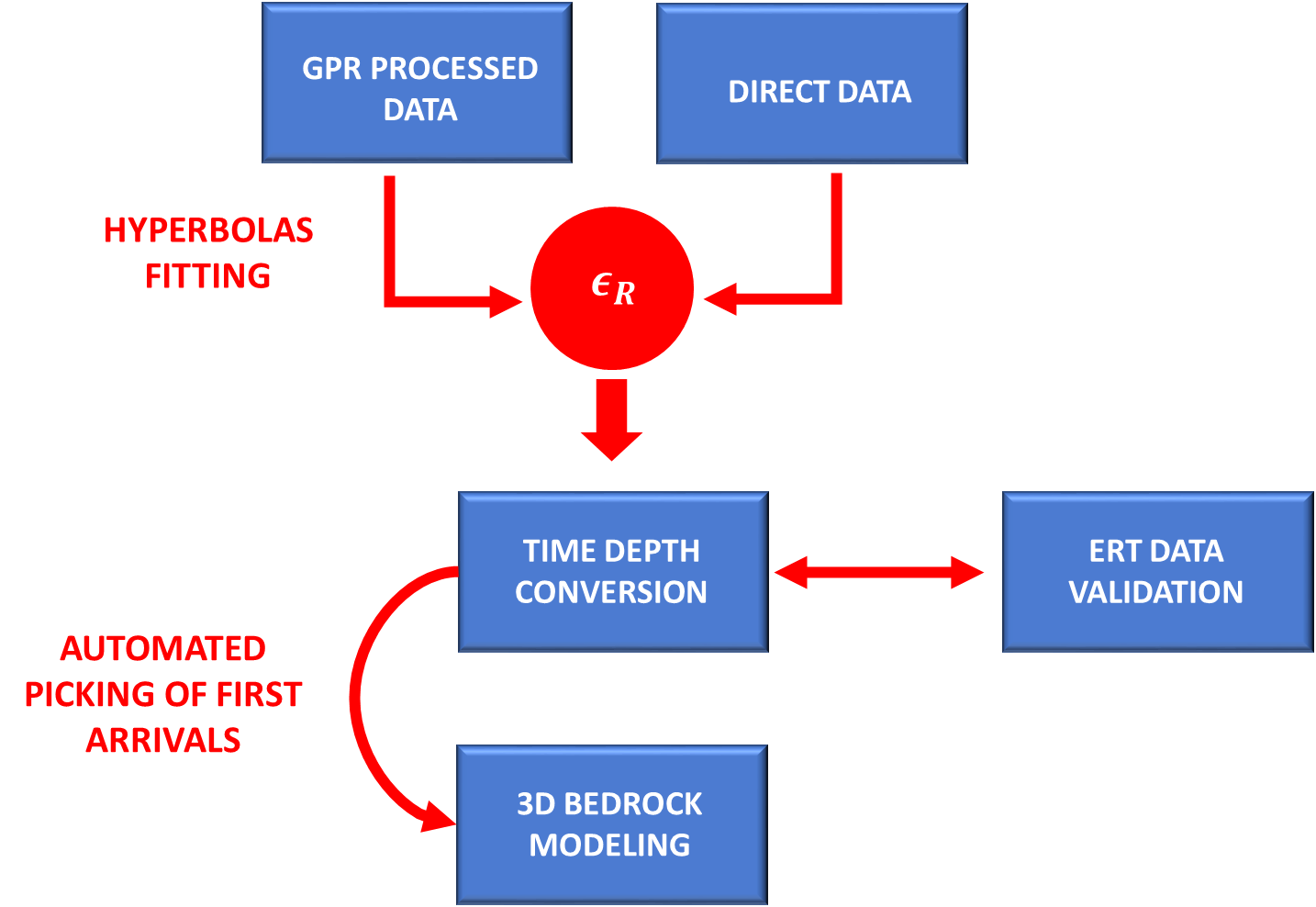


Figure S9. Customised procedure used to highlight the anomalies present in the GPR data.

Figure 3a, 3b, 3c, 3d show the comparison of the GPR and ERT acquired in the middle of the Plaza Principal and the strong agreement between the results obtained with the two techniques characterized by different resolutions and depths of investigation. Beyond the reflective layers characterizing the radargram in the first two meters of depth, related to different archaeological phases (see section 2.3.2), it is possible to identify a strongly reflective layer associable to the bedrock as confirmed by the ERT. This layer is well recognizable for most of its length, however near the west and east edges of the acquisitions some reflections seem to suggest the presence of discontinuities in the bedrock, presumably related to the quarry activities realized in the area (see Figure S10)


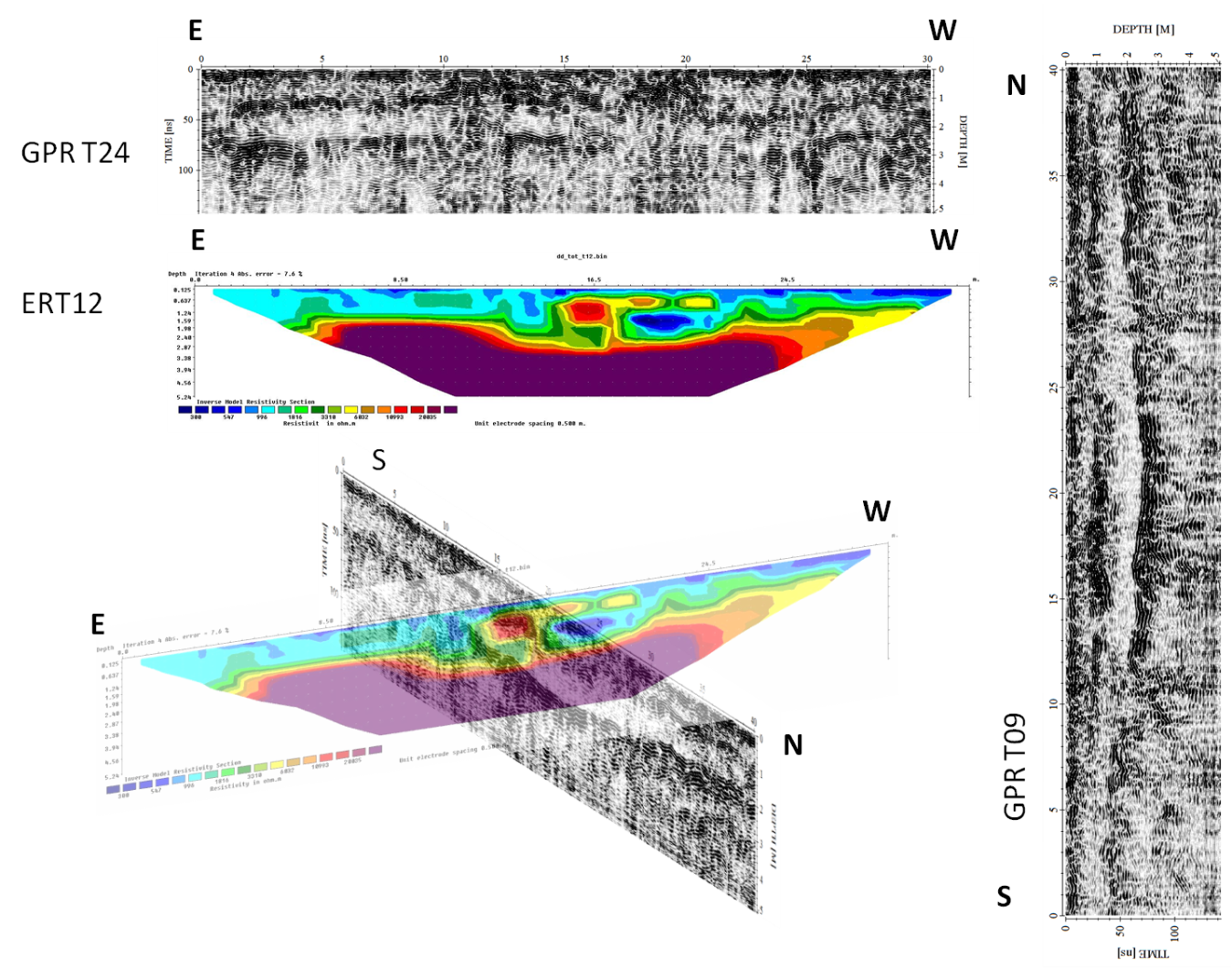


Figure S10 – ERT and GPR data integration which highlights the well fit of the results from the two geophysical techniques

**B.3 Geomagnetic prospections: method, data acquisition, processing, and results**

The geomagnetic method (MAG) is passive geophysical method widely used in a great variety of applications which range from small-scale investigation through to large scale geological analyses [67]. It is based on the mapping of local variations of the Earth magnetic field which result from changes of magnetic properties of the underlying rocks or from the presence of buried artifacts within the subsoil.

In archaeological explorations magnetic survey is one of the most important and widely applied geophysical techniques [68]. The possibility to scan large areas in a short time, the easy field operations and the relative intuitive data processing, make the MAG investigation an optimal tool for a preliminary survey of an area of potential interest. For the aforementioned reasons, it has been widely applied in archeology, from the small-scale site characterization to the surveying of large scale areas [69].

Magnetic surveys can be carried on by using different kind of sensors and by adopting different acquisition strategies (total field measurements, pseudo-gradiometric measurements) depending on the characteristics of the potential target [70]. For the archeological investigations, one of the most effective acquisition strategies is the pseudo-gradiometric (gradiometric one) one which consists in the simultaneous measurements of the magnetic field at two different position (height). Compared to the total field measurements, the vertical gradiometric measurement tends to better define shallower anomalies which are assumed to be of interest in the archaeological prospections and, for this reason, it is usually preferred to the former one. Furthermore, the gradiometric data are easier to process than the total field data not requiring operations like the regional field removal or the accounting for the diurnal variations of the magnetic field.

The measured field (both total or gradiometric) is the vector sum of the earth’s magnetic field and the field created by a local feature, the shape of the resulting anomaly depends on the geomagnetic latitude of the survey [70].

Local variations (anomalies) of the measured magnetic field can be related either to the induced magnetization or to the remanent magnetism. The induced magnetization is linked to the magnetization of a material as a response to the Earth’s Magnetic field and is a function of the magnitude of this last and of the magnetic susceptibility of the magnetized material. The remanent magnetism, on the other hand, is the results of several processes such as thermal or chemical alterations. In archaeological explorations, the induced magnetization is useful to detect variations in magnetic susceptibility between topsoil, subsoil and rocks. The remanent magnetization, on the other hand, makes possible to sense the presence man-made objects which were naturally heated or artificially as in the case of baked clay, pottery, etc. The range of magnetic anomalies generated by buried archaeological features spams form few nT (1–20 nT) for remains up to thousands of nT for fired structures (hearths, kilns, crockery, pottery) and ferrous objects [71].

In the *Plaza Principal* of Machu Picchu the MAG data here presented were acquired in gridded areas of various sizes (ranging from 20x20 m to 40x40 m) using survey procedure that are standards in archaeological prospection. The MAG data were collected using a Grad601-Bartington system. The system includes a data logger, a battery, two Grad-01-1000l sensors mounted on a rigid carrier bar. The instrumental sensitivity is ± 1 nT (nano Tesla). Calibration is performed on-site prior to acquisition through an automated procedure which allows us to correct possible misalignment in the sensors measurements. The gradiometer data were collected in unidirectional mode, along parallel profiles 1 m apart and at a 0.2 m sampling interval.

Standard processing procedures, using signal and image processing techniques, have been applied allowing the magnetic data to be rendered as an image. Finally, vertical gradient maps of the investigated areas were produced by applying a minimum curve interpolation (“spline”) to produce smooth magnetic maps.

Before commenting the magnetic survey results, some general comments are necessary.

- The Grad601-Bartington system only provides the measure of the vertical magnetic gradient and does not allow retrieving separately the upper and the bottom sensor measurements. This condition limits the possible data-modelling of the collected data and results in a mostly qualitative interpretation of the survey output.

- The vertical component of the magnetic intensity of the Earth’s magnetic field is a function of the latitude and reaches its minimum values at the magnetic equator. At Machu Picchu, at the time of the survey, the inclination of Earth’s magnetic ﬁeld was -40.53°, its declination -8.29° and its vertical component -11332 nT, it is hence expected a general reduction of the anomaly amplitudes.

- The most important single factor affecting detectability with a magnetometer is the distance between the magnetometer and the target object. If the survey area has an irregular surface, a constant height of the sensor from the surface cannot be guaranteed. Fluctuations of the vertical magnetic gradient may be hence generated.

- The bedrock of the Inca citadel of Machu Picchu is mainly composed by granite (magnetic susceptibility ranging from 0 to 50 x 10^3^ [72]. Soil cover, widely outcropping in the area, is mainly composed by individual blocks and subordinately by coarse materials originated by chemical and physical weathering of minerals. The terrace where the geophysical survey took place is generally composed of anthropogenic fill interrupted, in some points, by individual blocks of granite (i.e. sector E) whose presence may generate high vertical magnetic gradient anomalies.

The MAG map of the area E2 is characterized by a series of large amplitude dipolar anomalies (marked in figure S11 as “zone A”) and by one smaller amplitude but clearly visible feature (labelled as “B”).

The zone A coincides with an area which was subjected to archaeological excavation due to the presence of a granitic “monolite”. The results of the excavation (see sections 1, 2.3.2; and [23]) highlighted that, in past eras, the monolite was vertically placed and surrounded by “rural walls”, now mostly collapsed, and was a ritual place, as testified by the findings of small pieces of ceramics and carbons. The general aspect of the magnetic map, in the zone A resembles what above described with the smallest dipolar anomalies, mainly located on the edge of the excavated area, possibly related either to recent anthropogenic debris linked to the excavation activities (i.e. nails) or to the presence of ceramic and carbon debris. Another strong magnetic anomaly is related to the presence of a steel plate of GPR point.

Feature B is a circular anomaly which extends over the whole map. Due to its low amplitude, it may be related to the presence of a structural trench (such as a circular basin) or to an anthropogenic circular ditch.


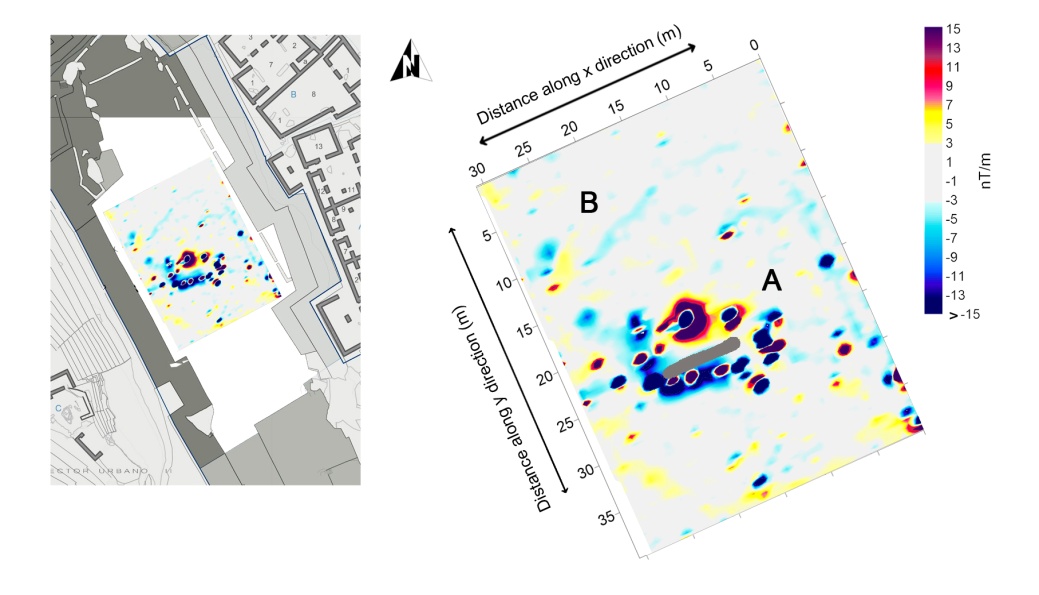


Figure S11 Magnetic results obtained in the Plaza Principal. (Left) Magnetic results overlaid on the architectual plan. (right) Zoom of the MAG map. The capital letters "A" and "B" indicate the position of the anomalous zone described in the main text. The wanka position is reported by the grey elongated body in the anomalous zone A..

**C Geological and Geomorphological analysis**

The monumental complex of Machu Picchu has a surface area of approximately 326 km^2^. It is located on the top of a graben-like structure at 2.430 m.a.s.l., in the high Eastern Cordillera of the Peruvian Andean chain, and 500 meters above the Vilcanota river (knew also as *Urubamba*) that cuts the Cordillera, approx. 110 km from Cuzco (Peru) (Fig. 1c). The Machu Picchu granitoid pluton, forming part of the larger “Quillabamba granite”, is one of a series of plutons intruded along the axial zone of the high Eastern Cordillera Permo-Liassic rift system (Fig. S12) including a variety of rock types, dominantly granites and granodiorites [72]. The bedrock of the Inca citadel of Machu Picchu is mainly composed by granite and subordinately granodiorite. The latter is mainly located in the lower part of the slopes (magmatic layering at the top). Locally, dikes of serpentine and peridotite are outcropping in two main levels; the former is located along the Inca trail, near Cerro Machu Picchu (vertically dipping), the latter is located along the path toward “Templo de la Luna” in Huayna Picchu relief. Superficially, the granite is jointed in blocks with variable dimensions, promoted by local structural setting and weathering. More in detail, local substrate often consists of large irregularly-piled granite boulders (granitic chaos) over which the urban area was built. Soil cover, widely outcropping in the area, is mainly composed of individual blocks and subordinately coarse materials originated by chemical and physical weathering of minerals. Part of the slopes exhibit debris accumulation as a result of past and present landslide activity [73]. Talus and talus cones are composed of fine and coarse sediments, depending on the local relief energy [73]. The dimension of single blocks is variable from 10^–1^ to about 3~10 m^3^. Grain size distributions of landslide accumulation are closely related to movement types and evolution [2].


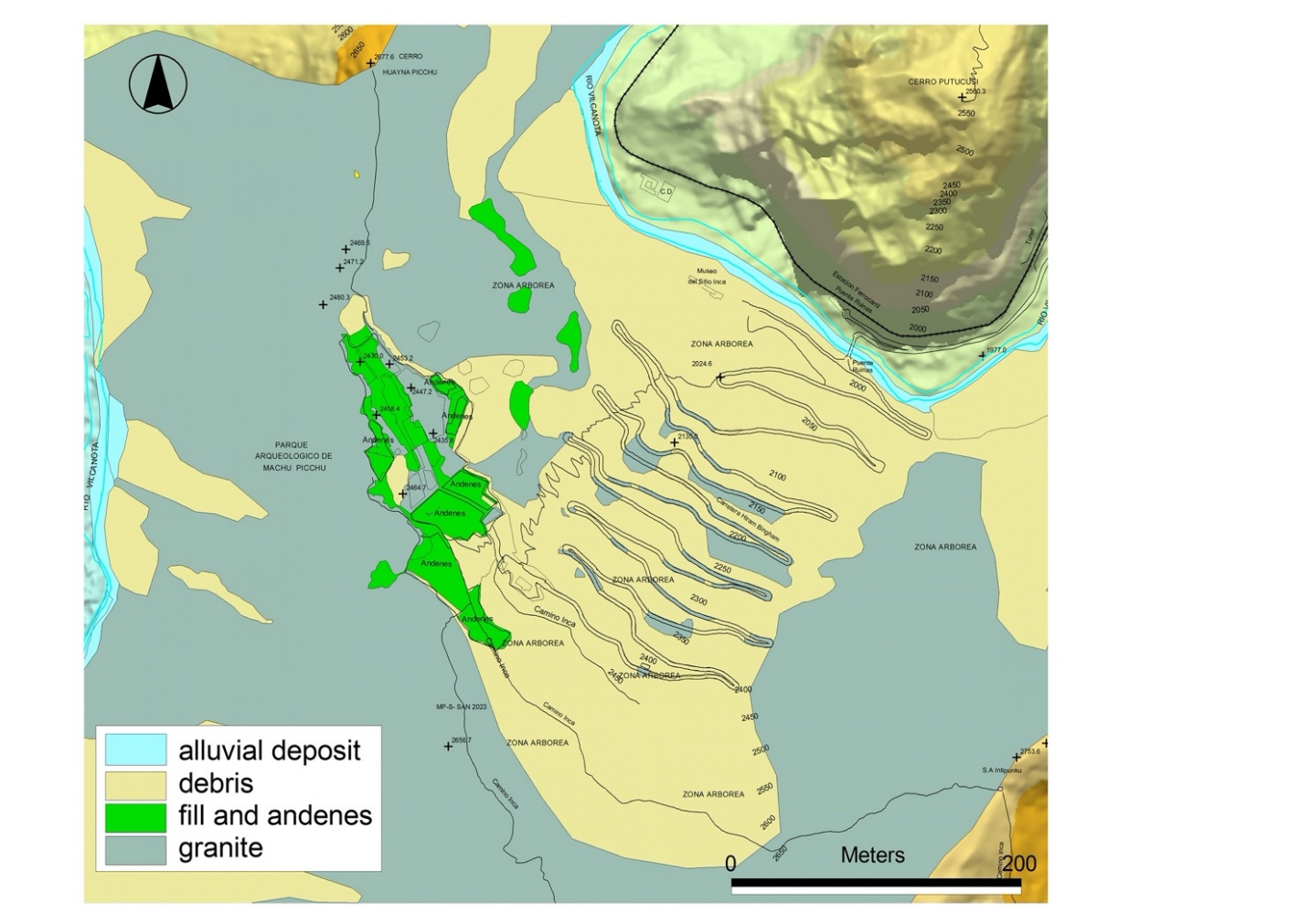


Figure S12. Detailed geological map developed on GIS platform (modified from Margottini et al., 2008).

From morphological point of view, alluvial deposits outcrop along allt the Vilcanota River and its tributaries. They are composed by heterometric and polygenic sediments that may be in lateral contact with the talus deposits. Anthropogenic fill and *andenas*, on top of Citadel (mainly present on the top of the *llaqta*), reflect the work of Inca urbanisation and civilization in the area. Besides chemical weathering, past and present mechanical processes such as crioclastism, thermoclastism and gravitational phenomena (mainly acting on steepest rock slopes) produce thick and extensive debris and talus/alluvial cones in the whole archaeological area. Very often, these types of soils are remobilised from their original place due to present and past morphogenetic processes and often triggered by heavy rainfall events. The continuous occurrence of morphological processes, both spatial and temporal, brought about the present morphology of the citadel (74). The bedrock evolution through granitic chaos formation [75] widely diffused in the study area, could be interpreted as one of the main reasons which led to the choice of the site as a perfect place for the construction of the citadel (e.g. availability of construction material). Several slope instability phenomena have been identified and classified according to mechanism, material involved and state of activity [76]. They are mainly related to rock falls, debris flows, rock slides and debris slides. Origin of phenomena is kinematically controlled by structural asset and relationship with slope face (rock falls, rock slide and debris slides); the so accumulated materials are the main source for debris flow. The situation of the slope with the citadel is more complex due to the strong structural control of the master joints on the slope evolution. In this, planar rock slides are mainly affecting the NE flank while rock falls are predominant on SE cliff [74]. The original geomorphological settings and context of the whole hill (before the arrival and action of the Inca) was characterized by an alternation of small basins characterized by a young drainage network in rapid evolution and by the presence of an alterated layer of the fractured granite bedrock. This is generally considered as the evolution of the bedrock in the so-called “ *granite chaos*” due to the succession of intense pluviometric phenomena associated (where geo-mechanically possible) with deepening in the head areas and deposition of blocks (various sizes) in the closing areas (small rock delta).

**D Archaeological record**

A Graphical summary of the excavations unit UE25, including the test pit 01, whose results have been discussed in sections 1 and 2.3.2, is showed in this paragraph (see Figures S13 and S14).

Figure 24 depicts the 3d overview of the investigated area with the localization of the GPR sections and results along with a vertical section of the scene which includes a GPR profile with the localization of UE25.

**
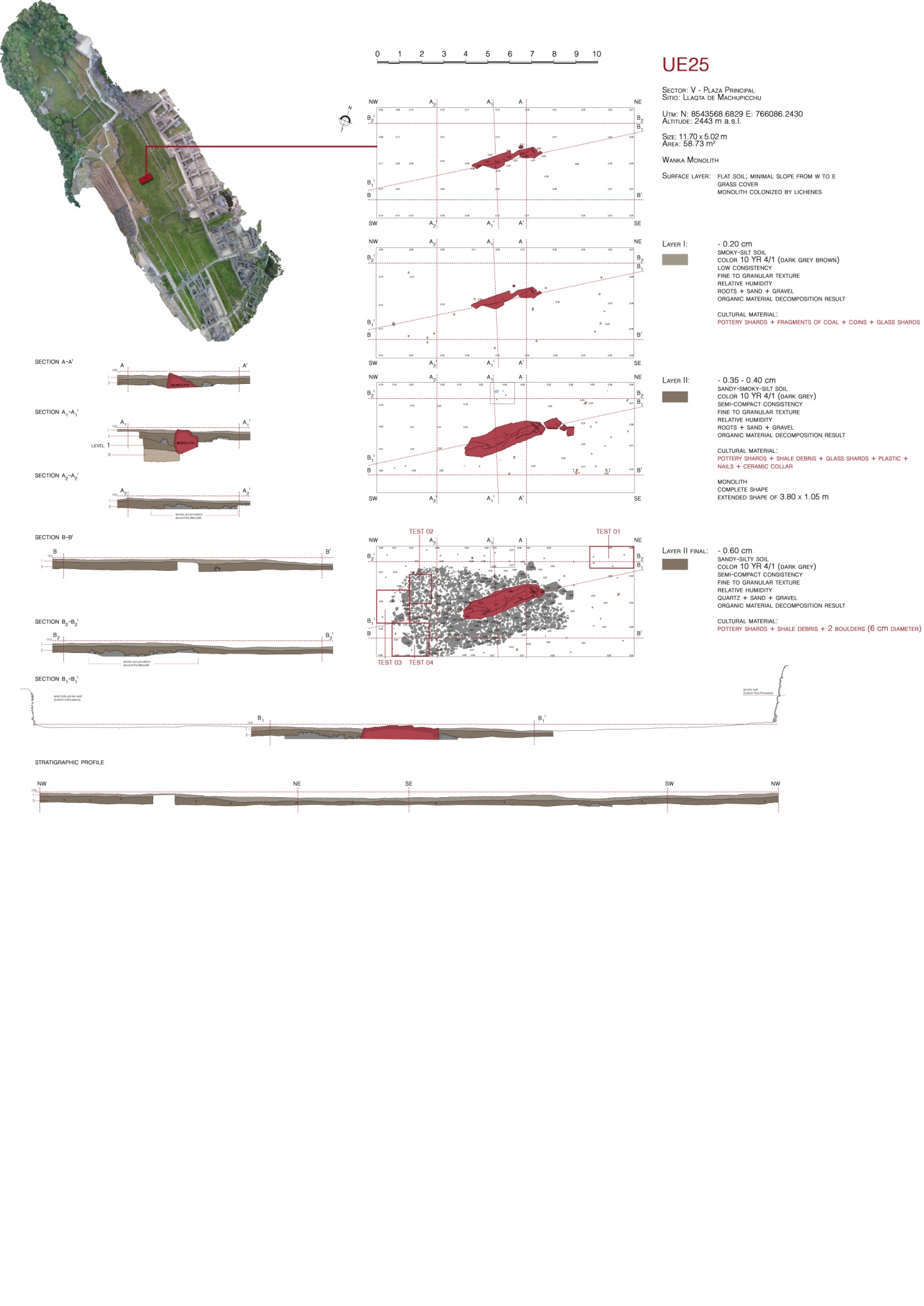
**

Figure S13. Graphical overview of the results obtained from excavation unit UE25 [23]

**
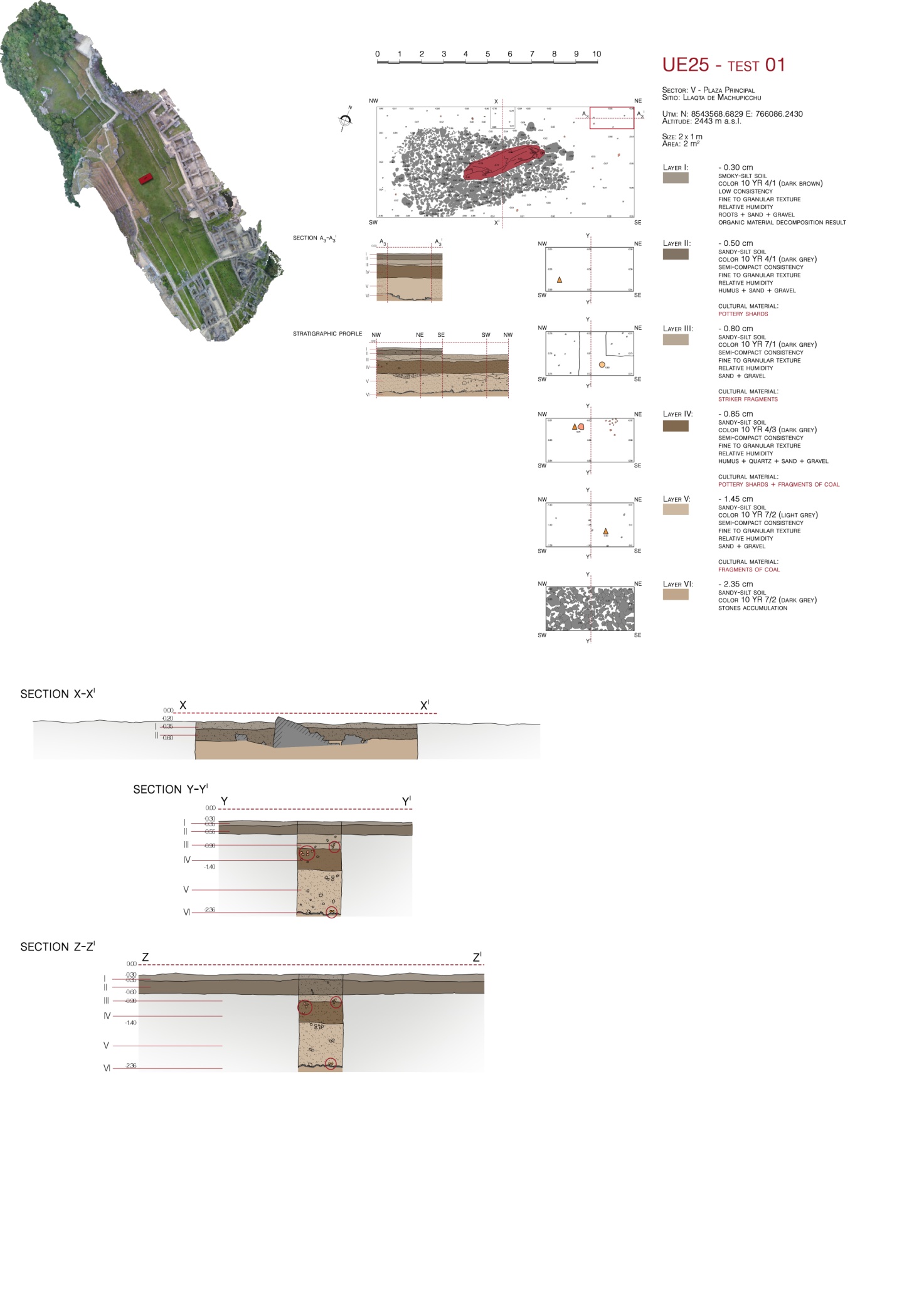
**

Figure S14. Graphical overview of the results obtained from test 1 of the excavation unit UE25 drawn from [23]

**
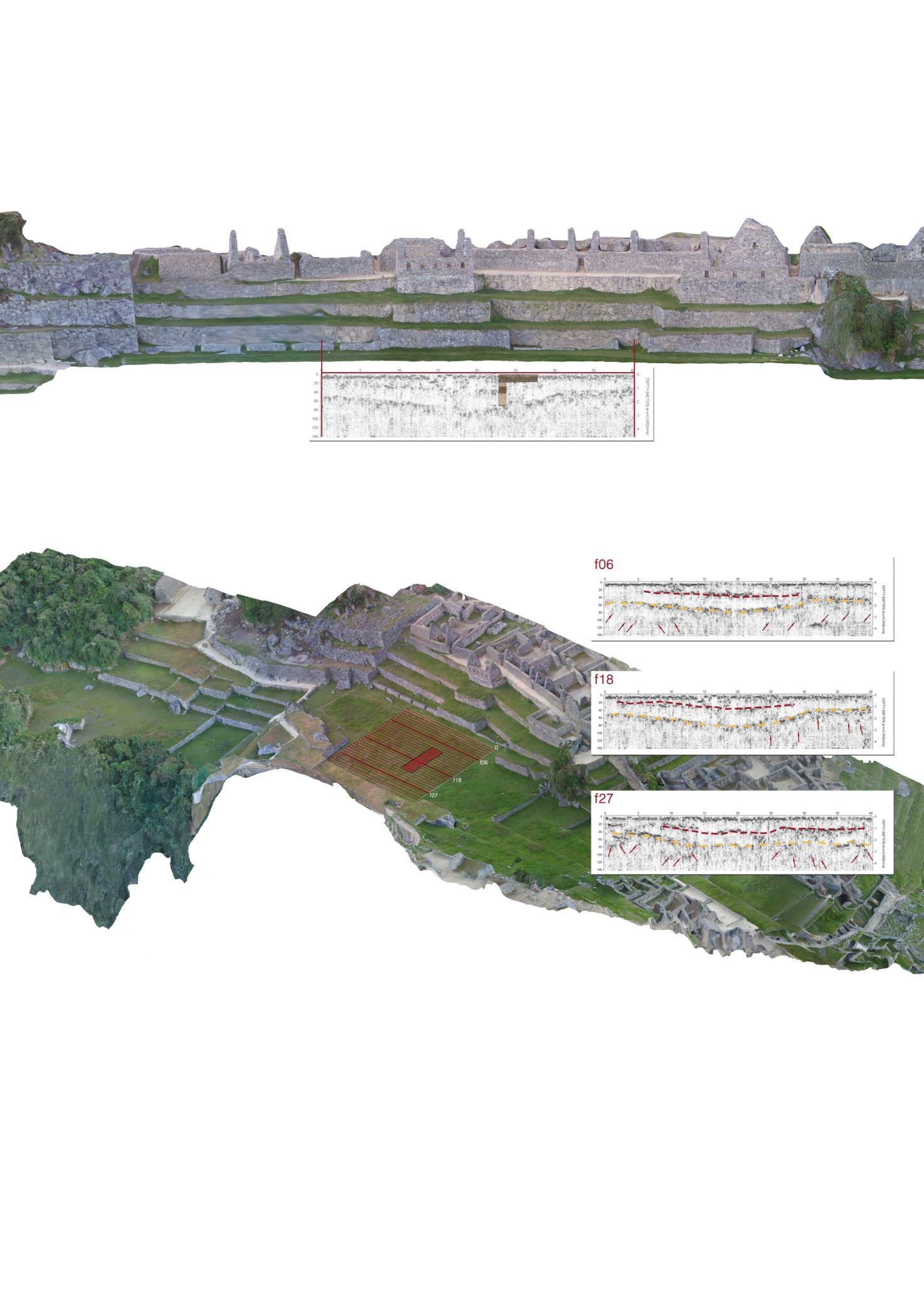
**

**
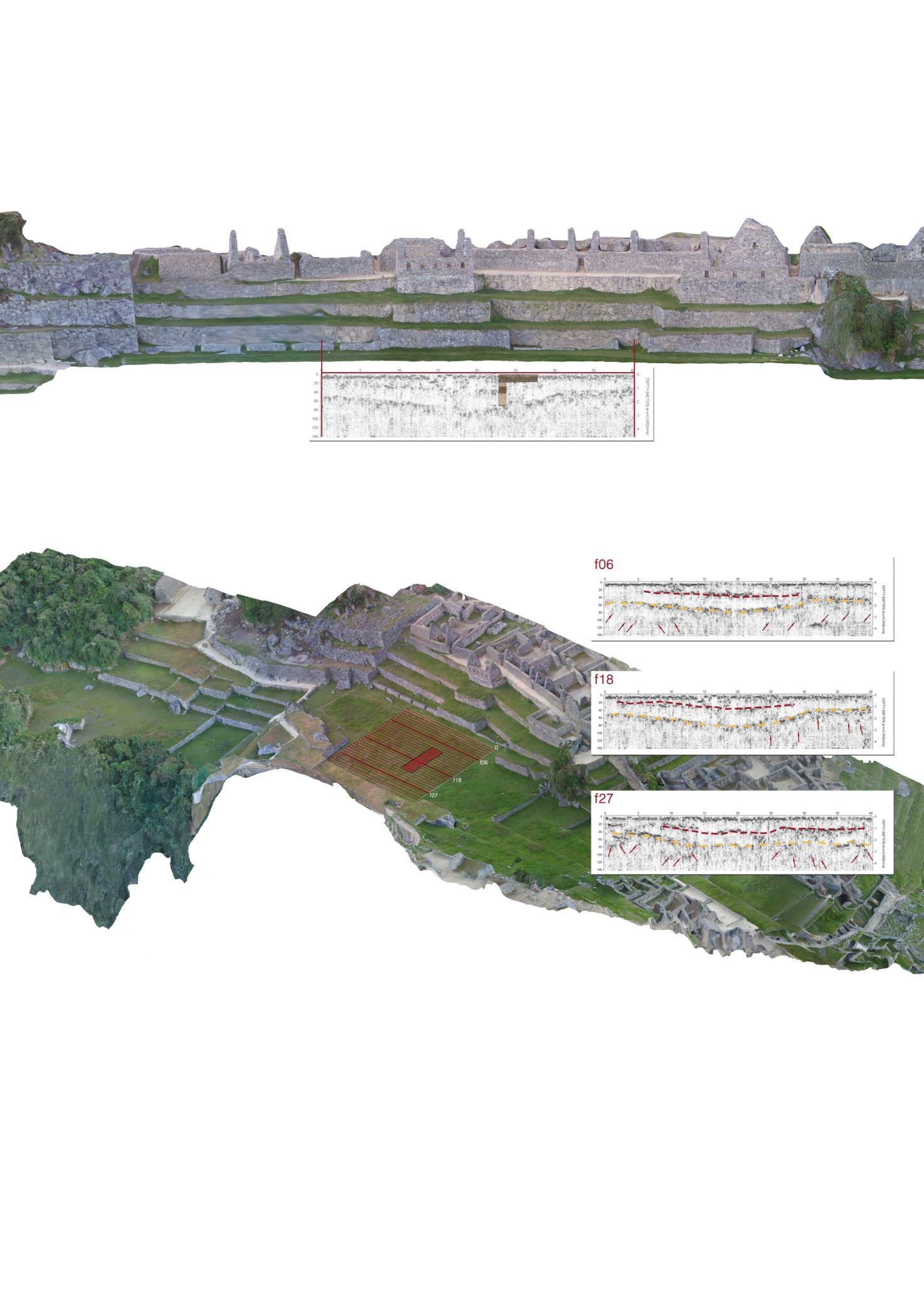
**

Figure S15. (up) 3d overview of the investigated area with the localization of the GPR sections and results. (bottom) Vertical section of the scene including a GPR section with the localization of UE25.

**REFERENCES**

See References in the manuscript
